# Nasal microbiota evolution within the congregate setting imposed by military training

**DOI:** 10.1038/s41598-022-15059-z

**Published:** 2022-07-07

**Authors:** Faith C. Blum, Jeannette M. Whitmire, Jason W. Bennett, Patrick M. Carey, Michael W. Ellis, Caroline E. English, Natasha N. Law, David R. Tribble, Eugene V. Millar, D. Scott Merrell

**Affiliations:** 1 Department of Microbiology and Immunology, Uniformed Services University of the Health Sciences, 4301 Jones Bridge Road, Bethesda, MD 20814 USA; 2 Walter Reed Army Institute of Research, Silver Spring, MD USA; 3 Benning Martin Army Community Hospital, Fort Benning, GA USA; 4 University of Toledo Medical Center, Toledo, OH USA; 5 Infectious Disease Clinical Research Program, Department of Preventive Medicine and Biostatistics, Uniformed Services University of the Health Sciences, Bethesda, MD USA; 6 Henry M. Jackson Foundation for the Advancement of Military Medicine, Inc., Bethesda, MD USA

**Keywords:** Microbiology, Microbiome

## Abstract

The human microbiome is comprised of a complex and diverse community of organisms that is subject to dynamic changes over time. As such, cross-sectional studies of the microbiome provide a multitude of information for a specific body site at a particular time, but they fail to account for temporal changes in microbial constituents resulting from various factors. To address this shortcoming, longitudinal research studies of the human microbiome investigate the influence of various factors on the microbiome of individuals within a group or community setting. These studies are vital to address the effects of host and/or environmental factors on microbiome composition as well as the potential contribution of microbiome members during the course of an infection. The relationship between microbial constituents and disease development has been previously explored for skin and soft tissue infections (SSTIs) within congregate military trainees. Accordingly, approximately 25% of the population carries *Staphylococcus aureus* within their nasal cavity, and these colonized individuals are known to be at increased risk for SSTIs. To examine the evolution of the nasal microbiota of U.S. Army Infantry trainees, individuals were sampled longitudinally from their arrival at Fort Benning, Georgia, until completion of their training 90 days later. These samples were then processed to determine *S. aureus* colonization status and to profile the nasal microbiota using 16S rRNA gene-based methods. Microbiota stability differed dramatically among the individual trainees; some subjects exhibited great stability, some subjects showed gradual temporal changes and some subjects displayed a dramatic shift in nasal microbiota composition. Further analysis utilizing the available trainee metadata suggests that the major drivers of nasal microbiota stability may be *S. aureus* colonization status and geographic origin of the trainees. Nasal microbiota evolution within the congregate setting imposed by military training is a complex process that appears to be affected by numerous factors. This finding may indicate that future campaigns to prevent *S. aureu*s colonization and future SSTIs among high-risk military trainees may require a ‘personalized’ approach.

## Introduction

The composition of an individual’s microbiome varies widely depending on the sampled body site. Moreover, the microbial constituents that are present at various sites are influenced by a multitude of factors, including geographic location, lifestyle, ethnicity, culture, diet, and physical health^1–7^. Additionally, it is clear that the presence of particular bacterial species can influence the prevalence of other species that are found within the microbiome^4,8–10^. These influences can be negative or positive and can occur through a variety of mechanisms. For example, *Staphylococcus epidermidis*, a commensal component of the human skin microbiome, produces phenol soluble modulins (PSMs) that function in conjunction with host anti-microbial peptides to kill *Streptococcus pyrogenes* and *S. aureus*^11–13^. Similarly, within the nasal cavity, members of the microbial community appear to prevent the establishment of other species; in vitro studies have shown that *Corynebacterium pseudodiphtheriticum* inhibits the colonization efforts of *S. aureus* and exhibits bactericidal activity against the pathogen^14–16^. Additionally, *C. accolens* produces free fatty acids that inhibit growth of *Streptococcus pneumoniae* within the nasal cavity^15,17^. Another skin commensal organism, *Corynebacterium striatum*, has a demonstrated ability to modulate *S. aureus* gene expression and elicit the transformation of the microbe from a virulent state to that of a commensal^18,19^. Conversely, some microbes cooperate with each other to promote colonization and sustained survival. For example, the presence of *C. accolens* correlates with *S. aureus* carriage in the nasal cavity and can enhance growth of *S. aureus *in vitro^15–17^. Likewise, the gut microbe, *Bacteroides ovatus*, induces the extracellular digestion of inulin to feed other species, such as *B. vulgatus*, within the intestinal tract^20^. This relationship between the species within the intestines has reciprocal benefits and actually increases the fitness of *B. ovatus*^20^. Thus, the interplay among the constituents of the microbial community is complex, and each member can positively or negatively influence the presence of other species within the microbial milieu.

While a great deal of information has been gained from cross-sectional studies that define the microbiome of a particular site at a particular time, the composition and relative abundance of various species within the microbiome of an individual can change over time. These changes can be driven by host and/or lifestyle factors^1–7^ as well as via simple exposure as an individual encounters other bacterial species within an environment and/or through interaction with others^2^. As such, the dynamic changes that occur within the human microbiome are being examined via longitudinal research studies that seek to examine the impact of environmental factors and/or personal interactions on the microbiome of individuals over a period of time. For example, longitudinal analysis of the gut microbiome revealed the influence of daily dietary changes on microbial composition; stability was linked to a more diverse diet^21^. Similarly, a longitudinal examination of the skin microbiome in adults revealed relative stability despite exposure to various environmental microbes^22^. In contrast, analysis of U.S. Air Force Academy cadets revealed an increase in the similarity of skin microbiomes, but not gut microbiota, among roommates upon cohabitation^23^. Finally, analysis of the nares of healthy individuals indicated a temporal stability in microbial composition as well as a lack of convergence of the nasal microbiomes among cohabiting couples^8^. Thus, longitudinal analyses of the human microbiome generate results that vary depending on the body site and cohort under examination.

The particular composition of an individual’s microbiome, in combination with host and environmental factors, can precipitate pathogenesis of commensal microorganisms and can result in infections. Furthermore, resident microbes can influence the ability of an encountered pathogen to colonize and cause disease^11,18,24–27^. The relationship between the presence of particular microbes and disease development has been specifically examined for skin and soft tissue infections (SSTIs), which are frequently encountered maladies within the Military Health System^28–30^. Due to the close quarters and frequent physical contact associated with military training, the rate of SSTIs (abscesses, cellulitis, folliculitis, etc.) is especially high among military trainees^28,30–33^. This high prevalence of SSTIs greatly impacts military readiness and creates an economic burden on the Military Health System^30^.

Even though *S. aureus* benignly colonizes the nares of approximately 25% of the population at any one time^34,35^, colonized individuals are known to be at increased risk for subsequent infections^35–38^. As such, *S. aureus* is the most frequently identified infectious agent within cutaneous abscesses^39,40^. Various studies have sought to identify links between the composition of the nasal microbiome, *S. aureus* colonization, and subsequent SSTI presentation among military recruits^32,41–44^. For example, among a cohort of military recruits presenting with SSTIs, colonization and infection with methicillin-resistant *S. aureus* (MRSA) appeared to be associated with a single acquisition event, thus underscoring the potential for host decolonization and environmental disinfection as strategies to prevent SSTIs in high-risk recruits^43^. In contrast, a separate *S. aureus* genomic analysis in two cohorts of high-risk recruits identified multiple transmission events of MRSA within platoons^45^. These two seemingly disparate analyses highlight the need to examine the microbiomes of individual recruits within a platoon to explore the potential exchange of microbes within the congregate setting experienced during military training. Moreover, given that individuals found in other congregate settings (prison inmates, athletic team members, residents of long-term care facilities, children attending daycare) are also at increased risk for SSTI development^46–51^, a greater understanding of microbiome evolution within the congregate setting may shed light on possible paths to prevent subsequent infections.

Herein we investigated the evolution of the nasal microbiome of a single platoon of male U.S. Army Infantry trainees stationed at Fort Benning, Georgia. Individuals were sampled longitudinally within 24 h of their arrival at Fort Benning until completion of their training 90 days later. Given the congregate setting and uniform diet, schedule, and environmental exposures encountered by these military trainees, we originally hypothesized that we would observe an overall convergence of the individuals’ nasal microbiota to a more similar composition. However, microbiota stability varied dramatically on an individual basis; some subjects showed great stability, some subjects showed gradual temporal changes and some subjects showed dramatic shifts in nasal microbiota composition. Analysis of available patient metadata suggests that *S. aureus* colonization and geographic origin of the trainees may be key drivers of nasal microbiota stability within this population.

## Results

### Participant characteristics

A total of 627 male trainees were enrolled from 2015–2016 in an observational cohort study of *S. aureus* colonization and SSTI^52^. Participants were recruited from four separate Infantry training companies (two in 2015 and two in 2016), each composed of ~ 200 trainees. To determine the evolution of the nasal microbiota of individuals within the congregate setting imposed by infantry training, 53 male subjects from a single platoon enrolled in 2015 were analyzed. Demographic information for the subjects is provided in Table 1. Briefly, the median age was 19 years (range 17–34), the majority were non-Hispanic whites (37), and the individuals originated from various U.S. locations; approximately half of the subjects were from the South (26), followed by subjects from the Midwest (12), the Northeast (10), and the West (5). Metadata for individual subjects is provided in Supplementary Table S1.

**Table 1:** Study subject characteristics.

**Median age (range)**	19 (17–34)
**Race/ethnicity**	
Non-Hispanic/Latino	
White	37
Black or African American	5
Two races (White/Black or African American)	2
Not reported	2
Hispanic/Latino	
White	5
Not reported	2
***S. aureus* nasal colonization**^1^	
Non-colonized	20
Intermittent	22
Persistent	9
NA^2^	2
**Geographic origin**^3^	
Midwest	12
Northeast	10
South	26
West	5

^1^*S. aureus* nasal colonization was defined based on culture-positive *S. aureus* in the nares over the five study visits: non-colonized (positive at 0 study visits), intermittent (positive at ≥ 1 and ≤ 3 study visits), or persistent (positive at ≥ 4 study visits).

^2^Was not assessed for the two subjects that did not complete the five study visits.

^3^Based on zipcode listed on intake forms upon arrival. Regions as defined by US Census Bureau.

### Nasal swabs and culture analysis

**Figure 1:**
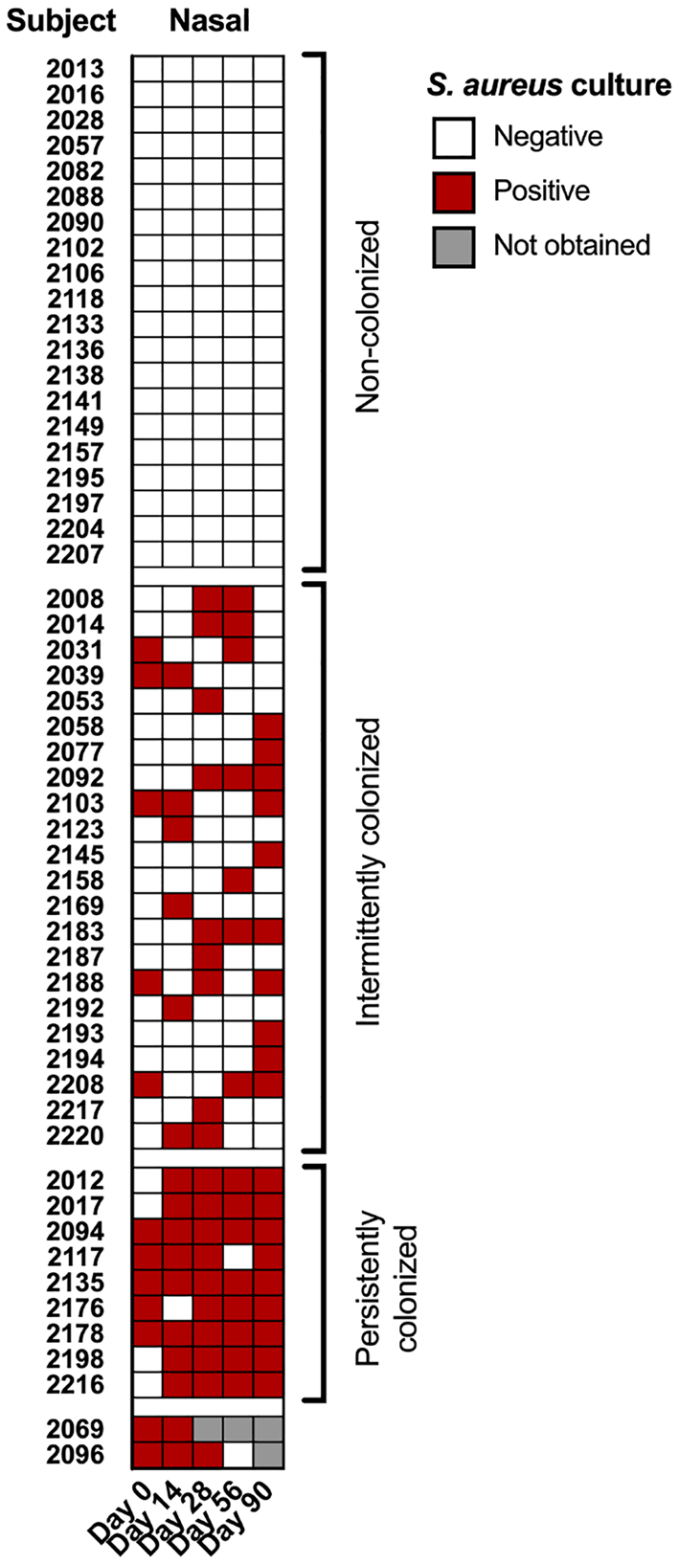
*S. aureus* culture status in the nares. Nasal swabs were collected upon arrival of subjects at Fort Benning (day 0) and then longitudinally at days 14, 28, 56 and 90. The swab was immediately used for *S. aureus* culture. Each trainee is listed by subject ID number, and the culture results for each day are shown. Positive culture results are denoted by red boxes, and negative culture results are indicated by white boxes. Results were not obtained for two subjects on different days, and those results are indicated by gray boxes. Trainees are categorized into general groupings based on *S. aureus* colonization status: not colonized (no positive cultures), intermittently colonized (≥ 20% and < 80% positive cultures), or persistently colonized (≥ 80% positive cultures). The two subjects that did not complete all 5 study visits are not categorized.

Paired nasal swabs were collected by the study staff upon arrival of subjects at Fort Benning (day 0) and then longitudinally at days 14, 28, 56 and 90. On each day, one swab was immediately used for *S. aureus* culture, while the second swab was stored at − 80 °C for future microbiota analysis. The day 0 swabs were used to define the baseline *S. aureus* nasal culture status/nasal microbiota. The subsequent swabs were used to identify any changes in *S. aureus* colonization status/structure and composition of the nasal microbiota. A total of 12/53 trainees (22.6%) were culture positive for *S. aureus* at day 0 (Fig. 1). While culture positivity varied across the study, overall colonization was increased at the end of training. A total of 19/51 trainees (37.2%) were culture positive at day 90; two subjects did not complete all 5 study visits and a day 90 sample was not available for these individuals. Overall, of the obtained 261 culture swabs, 81 were *S. aureus* positive (Fig. 1). *S. aureus* nasal colonization among individuals is known to fall into three broad categories: persistent carriers, intermittent carriers and persistent non-carriers. Thus, trainees were categorized into these general groupings based on the following definitions: not colonized (no positive cultures), intermittently colonized (≥ 20% and < 80% positive cultures), or persistently colonized (≥ 80% positive cultures) (Fig. 1). The two subjects that did not complete all 5 study visits could not be categorized. A total of 20 subjects (39.2%) were not nasally colonized by *S. aureus*, 22 subjects (43.1%) were intermittently colonized, and 9 subjects (17.6%) were persistently colonized. These numbers are in line with other studies concerning the prevalence of *S. aureus* in the nares^16,35,53,54^. At the individual level, we observed a variety of colonization patterns. Of the 22 intermittently colonized subjects, 12 were positive at only a single study visit. Of these, 7 were positive at one study visit, and negative at all following visits. Even among the 9 persistently colonized subjects, only 3 subjects were positive at each visit (Fig. 1).

Over the course of the 3-month study, 2 subjects developed a purulent SSTI: Subject 2013 on day 94; and Subject 2197 on day 22. Nasal colonization by *S. aureus* is a known risk factor for SSTI development^35,37,38^. However, neither subject was colonized by *S. aureus* in the nares at any of the sample timepoints.

### Microbiota sequencing

To analyze temporal changes in the nasal microbiota, the remaining set of paired nasal swabs was subjected to DNA extraction. To reduce the likelihood of bias, the 261 swabs were randomly arrayed across three 96-well plates for the extraction. Additionally, each plate contained one well with a mock community (positive extraction control), and 2 or 3 wells containing no input (negative extraction control). Extracted DNA was used for amplification and MiSeq-based sequencing of the V1-V3 region of the rRNA gene; positive and negative sequencing controls were also included in the sequencing reactions. One sample (Subject 2103 Day 90) yielded no sequence data, even after multiple attempts; the remaining 260 subject samples yielded a total of 18,135,243 raw sequences, with an average of 69,751 sequences per subject. The three positive DNA extraction controls yielded 966,548 sequences (mean, 322,183). Similarly, the three positive sequencing controls generated 368,129 sequences (mean, 122,710) sequences. In contrast, the eight negative DNA extraction and three negative sequencing controls yielded 6299 sequences (mean, 787) and 12,808 sequences (mean, 4269), respectively.

Forward and reverse sequencing reads were trimmed and assembled using FLASH^55^. Assembled sequences were analyzed using the R Studio implementation of DADA2^56,57^. After quality filtering and chimera removal, a total of 10,265,661 sequences from subject samples remained (56.6%), representing an average of 39,483 sequences per sample. From the control samples, a total of 355,294 (positive extraction controls), 142,401 (positive sequencing controls), 787 (negative extraction controls), and 96 (negative sequencing controls) sequences remained. The sequences assembled into a total of 12,359 amplicon sequence variants (ASVs); these were aligned to the Silva database, with species designations added where possible. The percent abundance of each ASV for each sample is provided in Supplementary Table S2. For downstream analyses, the control sequences were removed. After processing, three trainee samples had fewer than 1000 sequences (Subject 2014 Day 28, Subject 2149 Day 28, and Subject 2198 Day 90) and were removed from the downstream analyses.

### Microbiota characterization

**Figure 2:**
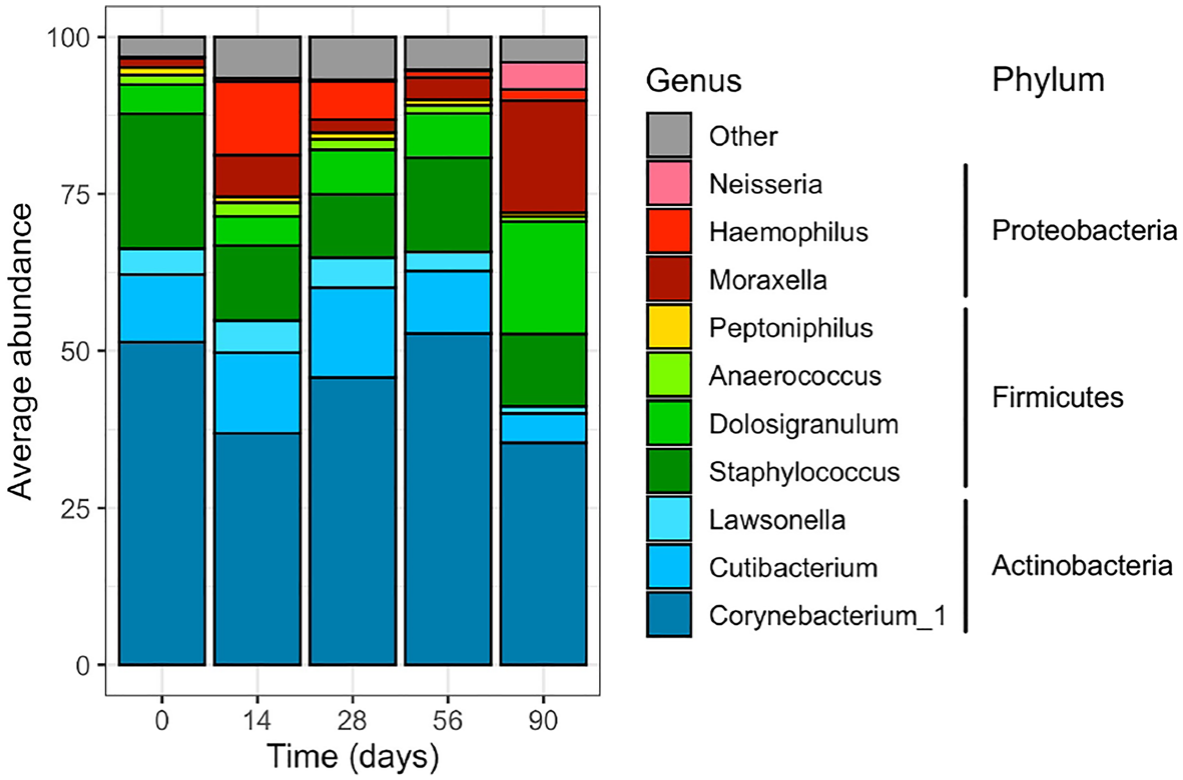
Distribution of ten most abundant genera across time. Nasal swabs were collected upon arrival of subjects at Fort Benning (day 0) and then longitudinally at days 14, 28, 56 and 90. The swabs were used to analyze the nasal microbiota of each subject. The sequencing results for the trainees are combined for each time point, and the average abundance of the ten genera that averaged ≥ 1% abundance is plotted across time.

The nasal microbiota of the subjects largely consisted of members of the Actinobacteria, Firmicutes, and Proteobacteria phyla. Ten genera averaged ≥ 1% abundance at any time point: *Corynebacterium_1*, *Cutibacterium*, and *Lawsonella* of Actinobacteria; *Staphylococcus*, *Dolosigranulum*, *Anaerococcus*, and *Peptoniphilus* of Firmicutes; and *Moraxella*, *Haemophilus*, and *Neisseria* of Proteobacteria. The abundance of these genera for each subject at each time point is shown in Supplementary Fig. S1. Based on a visual analysis, the microbiota of some subjects was relatively stable over time (e.g., 2141 and 2207); others changed drastically in composition, often with increases in Proteobacteria (e.g., 2008 and 2220); and other subjects showed steady shifts in their microbiota over time (e.g., 2096 and 2135). At the population level, these changes were visible when the average abundance of the ten genera that averaged ≥ 1% abundance were plotted across time (Fig. 2 and Supplementary Fig. S2). Broadly speaking, the abundance of Actinobacteria and *Corynebacterium_1* was unstable, and showed a biphasic pattern of changes; a decrease was observed at day 14, followed by increases at day 28 and 56 and another decrease at day 90. The abundance of Firmicutes was relatively stable across the study, but the contributing genera shifted; a decrease in *Staphylococcus* and an increase in *Dolosigranulum* were observed. Finally, the abundance of Proteobacteria represented a mirror image of the Actinobacteria changes; there was an increase at day 14, a decrease at days 28 and 56, and another increase at day 90. Of note, the genera contributing to these increases were different over time: *Haemophilus* and *Moraxella* at day 14, but *Moraxella* and *Neisseria* at day 90. Statistical analysis revealed that the temporal changes were significant for all of the highest abundance phyla and genera with the exception of *Anaerococcus* and *Peptoniphilus* (Supplementary Figs. S3, S4 and Supplementary Tables S3, S4). Overall, while there were observable differences in the nasal microbiota of the trainees, these changes occurred at different times and to a different extent for each trainee.

Given that changes differed dramatically across subjects, we hypothesized that host-specific factors influenced the observed stability/instability of the nasal microbiota. To this end, available metadata for each trainee was layered into the analyses. Specifically, we examined the contribution of nasal *S. aureus* culture (negative or positive), nasal *S. aureus* colonization status (persistent carriers, intermittent carriers and persistent non-carriers) and subject geographic origin (Midwest, Northeast, South, West). As expected from a prior study^41^, positive nasal *S. aureus* culture and persistent colonization status were associated with higher abundance of Firmicutes and *Staphylococcus* and lower abundance of Actinobacteria and *Corynebacterium_1* (Supplementary Figs. S3, S4 and Supplementary Tables S3, S4). The abundance of *Corynebacterium_1* was highest in subjects from the Midwest and lowest in subjects from the West, with the opposite observation for *Peptoniphilus*. Intermittent *S. aureus* colonization status was associated with an increase in Proteobacteria and *Moraxella* (Supplementary Fig. S4 and Supplementary Table S4). Further analysis by two-way ANOVA demonstrated that this was specifically due to the increase in Proteobacteria and *Moraxella* at day 90 among the intermittently colonized subjects (Supplementary Figs. S5, S6 and Supplementary Tables S5, S6).

To determine the genera that were differentially represented in the various groupings by an independent metric, LEfSe analysis was also utilized^58^. An advantage of this method is that it detects differences among genera of lower abundance. To this end, there were many genera that were differentially represented based on time (Supplementary Fig. S7A). Among the genera that were increased were *Lawsonella*, *Mycobacterium*, *Sphingomonas*, *Pantoea*, *Methylobacterium*, and *Pseudomonas* on Day 14; *Cutibacterium* and *Chryseobacterium* at Day 28; and *Dolosigranulum* and *Brachybacterium* at Day 90. The genera that were differentially represented based on positive or negative *S. aureus* culture (Supplementary Fig. S7B) and persistently colonized or not colonized by *S. aureus* were highly similar (Supplementary Fig. S7C); *Staphylococcus*, *Cutibacterium*, *Propionibacterium*, *Anaerococcus*, and *Peptoniphilus* were more highly represented in samples positive for *S. aureus* culture and in samples from persistently colonized samples. In addition, *Corynebacterium_1* and the family Neisseriaceae were more highly represented in trainees that were negative for *S. aureus* culture and in persistent non-carriers of *S. aureus*. *Moraxella*, *Dolosigranulum*, and *Raoultella* were highly represented in samples from intermittently colonized subjects. Finally, only two genera were differentially represented based on geographic origin of the subjects (Supplementary Fig. S7D): *Corynebacterium_1* in the Midwest and *Raoultella* in the Northeast.

### Alpha and beta diversity analysis

**Figure 3:**
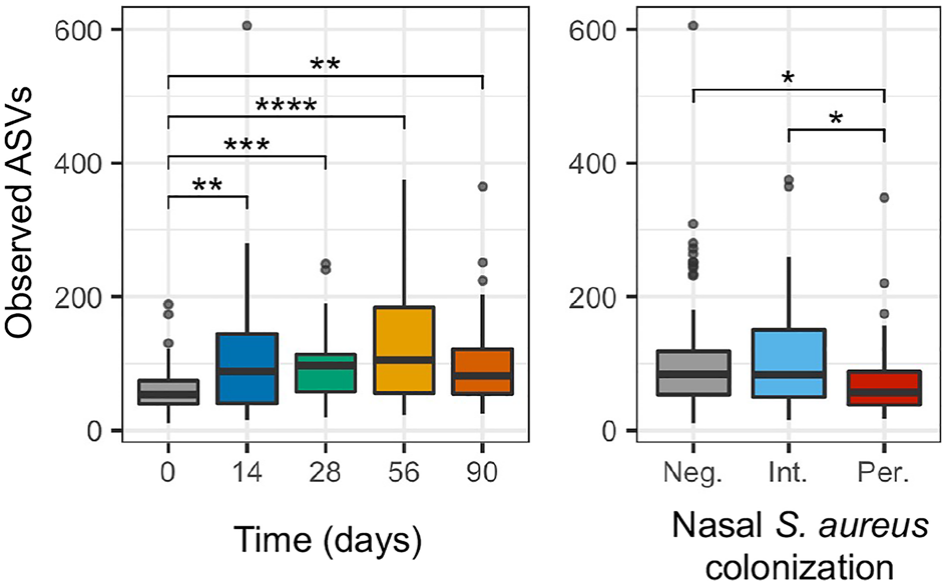
Measurement of alpha diversity. The number of observed ASVs are graphed as a measure of richness. The observed ASVs are displayed over time and based on *S. aureus* colonization status. *S. aureus* colonization status is noted as follows: negative (Neg.), intermittent (Int.), or persistent (Per.). For nasal *S. aureus* colonization, “NA” values are not graphed. Pairwise non-parametric Wilcoxon tests were performed with Benjamini and Hochberg correction for multiple comparisons. Statistical significance is indicated by asterisks (*): *P < 0.05; **P < 0.01; ***P < 0.001; and ****P < 0.0001.

Alpha diversity was measured using three common metrics: observed ASVs to measure richness, and Shannon diversity index and inverse Simpson index to measure diversity. The richness of the samples changed over time and based on the *S. aureus* nasal colonization category of the subject (Fig. 3 and Supplementary Fig. S8). The number of observed ASVs was lowest at day 0 (mean 63.9 ASVs) and increased over time, with a peak at day 56 (mean 146.6 ASVs) (*P* < 0.001, Supplementary Table S7). Subjects who were persistently colonized by *S. aureus* had fewer observed ASVs (77.4 ASVs) compared to those who were not colonized or were intermittently colonized (103.0 and 110 ASVs, respectively, *P* = 0.033) (Fig. 3). Neither diversity index changed across time or based on metadata (Supplementary Fig. S8 and Supplementary Table S7).

To further assess differences between the groups, beta diversity was assessed using the Bray–Curtis metric^59^, which is calculated based on the structure of the community. For this analysis, beta diversity is measured between each pair of samples, and a matrix of values is returned. The resulting distance matrix was visualized using principal co-ordinate analysis (PCoA) wherein samples that are most dissimilar are plotted the farthest apart, and samples that are more similar are plotted closer together. PCoA visualization of the temporal beta diversity by individual subject is provided in Supplementary Fig. S9. Some subjects were relatively stable over time; the points remained close to each other (e.g., 2145 and 2207). In contrast, many subjects showed substantial temporal changes (e.g., 2012 and 2220); the points were scattered across the PCoA plot. These individualized changes in beta diversity are in keeping with the trainee-specific differences in the abundance of phyla and genera highlighted above (Supplementary Fig. S1).

Beta diversity analysis combined with trainee metadata revealed unexpected interactions. Namely, distinct patterns were observed based on the geographic origin of the subjects; samples from the West and Northeast largely segregated to the upper quadrants and samples from the Midwest and South scattered throughout the plot (Supplementary Fig. S10). This finding was unexpected since only a few significant differences in the abundance of phyla and genera were observed when samples were grouped by geographic origin (Supplementary Figs. S3–S6). Thus, the differences elucidated by the beta diversity analysis are likely driven by the contribution of lower abundance phyla and genera to the structure of the microbiota.

**Figure 4:**
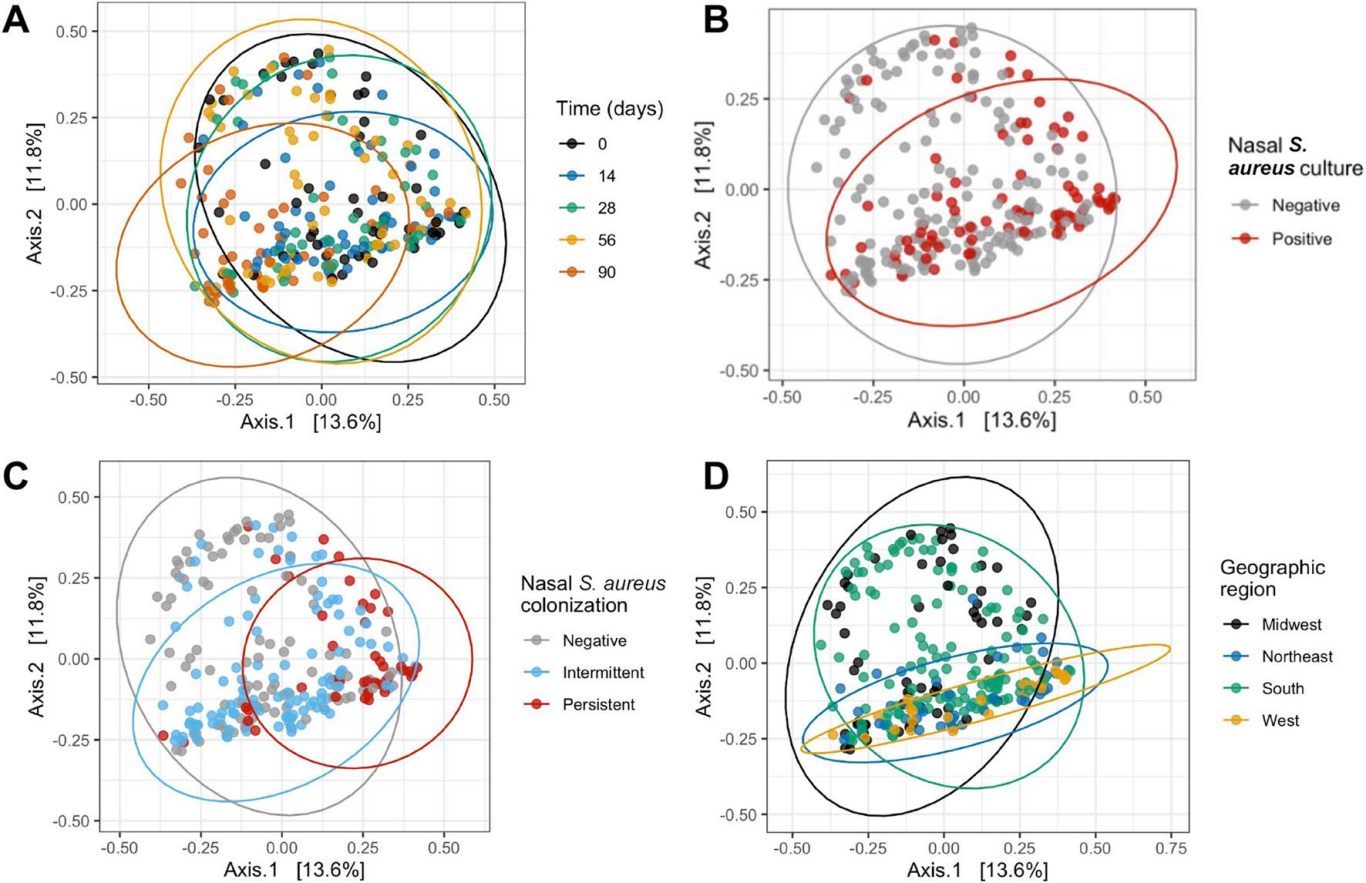
Principal co-ordinate analysis (PCoA). Bray–Curtis distance of the samples is visualized by PCoA based on time (**A**), nasal *S. aureus* culture (**B**), nasal *S. aureus* colonization (**C**), and geographic region (**D**). For nasal *S. aureus* colonization, “NA” values are not graphed. Bray–Curtis distances were ordinated by principal co-ordinate analysis (PCoA) using *phyloseq*, and graphed using *phyloseq* and *ggplot*.

Further examination of the beta diversity plot from the Northeast revealed that the five points located in the lower right-hand quadrant and distant from the other points from the Northeast samples, were from a single subject (2141). Reexamination of the participant questionnaires revealed that subject 2141 was the only subject in the platoon who self-reported the use of antibiotics in the 6 months prior to study enrollment. Thus, antibiotic use may be responsible for diversity differences observed in this individual. To eliminate this potential confounding factor, this subject was removed from downstream analysis. Indeed, upon removal of samples from this subject, the orientation of the PCoA shifted, rotating approximately 180°, with subjects from the Northeast and West now clustered in the lower quadrants of the plot (Fig. 4D).

Visual analysis of samples based on time, *S. aureus* culture, *S. aureus* colonization grouping, and geographic origin elucidated differences within groups (Fig. 4). For example, the day 14 samples were compressed into a smaller area than samples from days 0, 28, and 56. Furthermore, the 90 samples shifted toward the lower left quadrant of the plot (Fig. 4A). These observations suggest time-dependent oscillations in the nasal microbiota structure. While the microbiota at days 0, 28, and 56 were similar, a microbiota shift occurred at day 14. This shift was largely reversed by day 28. An additional shift occurred between days 56 and 90. Next, distribution of samples from *S. aureus* culture positive trainees were compressed as compared to the culture negative samples (Fig. 4B). Finally, samples from subjects who were intermittently colonized by *S. aureus* tended to cluster to the lower quadrants, and those from persistently colonized subjects were segregated to a smaller cluster in the lower right quadrant (Fig. 4C).

The statistical significance of these visual observations was next assessed using PERMANOVA analysis: time, *S. aureus* culture, *S. aureus* colonization status, geographic origin, and subject were all significant factors (Table 2). Pairwise PERMANOVA analysis further elucidated the relationship between individual factors (Supplementary Table S8). The microbiota at days 0, 28, and 56 were not significantly different from one another. However, day 14 was significantly different from every other time point except day 28, and day 90 was significantly different from every other time point. All samples from subjects that were not colonized, intermittently colonized, and persistently *S. aureus* colonized were significantly different from each other. Finally, the samples from each geographic origin were significantly different from the others.

**Table 2:** Bray–Curtis beta diversity distances were tested by PERMANOVA analysis. One-way comparisons were tested: time, nasal *S. aureus* culture, nasal *S. aureus* colonization, geographic region, and subject. For nasal *S. aureus* colonization, subjects with “NA” status were removed. PERMANOVA was performed using vegan’s adonis function^78^, using 999 permutations.

Parameter	Pr (> F)
Time	0.001
Nasal *S. aureus* culture	0.001
Nasal *S. aureus* colonization	0.001
Geographic region	0.001
Subject	0.001

**Figure 5:**
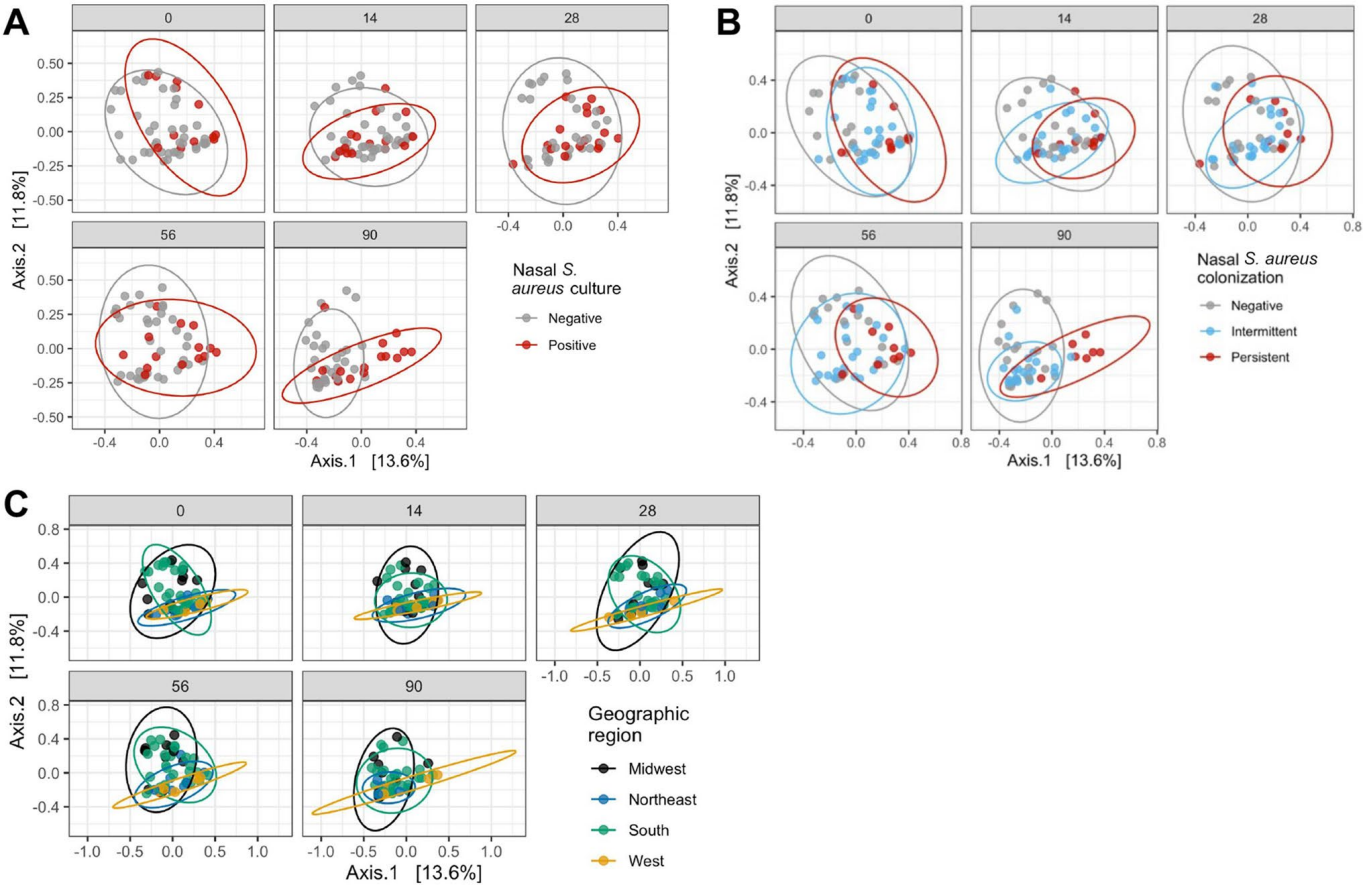
Principal co-ordinate analysis (PCoA) over time. Bray–Curtis distance of the samples over time is visualized by PCoA, colored by nasal *S. aureus* culture (**A**), nasal *S. aureus* colonization (**B**), and geographic region (**C**). For nasal *S. aureus* colonization, “NA” values are not graphed. Bray–Curtis distances were ordinated by principal co-ordinate analysis (PCoA) using *phyloseq*, and graphed using *phyloseq* and *ggplot*.

To understand the temporal impact of nasal *S. aureus* culture, nasal *S. aureus* colonization status, and geographic origin, each factor was assessed by sample time point (Fig. 5). The difference between samples from *S. aureus* culture negative or positive trainees were most apparent at the end of the study; *S. aureus* negative samples scattered throughout the plot at all time points, but samples from *S. aureus* positive individuals condensed into a smaller area by day 90 (Fig. 5A). Similarly, the difference between samples from subjects not colonized, intermittently colonized or persistently colonized by *S. aureus* was most apparent at day 90; at this point samples from persistently colonized subjects segregated in the lower quadrants and those from intermittently colonized subjects segregated in the lower left quadrant (Fig. 5B). In contrast, the samples from subjects from different geographic regions were different from one another at all study visits but were temporally consistent at all time points: those from the Northeast and West remained largely within the lower quadrant and those from the Midwest and South were scattered throughout the plot (Fig. 5C).

Finally, we examined the contribution of race and ethnicity to the observed beta diversity (Supplementary Fig. S11). Several groups were visually distinct from the others, especially the Non-Hispanic/Latino, White and Black and the Hispanic/Latino, Not reported. However, the low number of subjects that fell into each of these categories (2 subjects each for the categories listed above) limits any conclusions about nasal microbiota difference that may be attributed to race and ethnicity in this study.

### Longitudinal analyses

A major goal of this study was to test the hypothesis that the congregate setting would lead to an overall convergence of the individuals’ nasal microbiomes to a more similar composition over time. To measure these changes, the distribution of Bray–Curtis distance values between all pairs of subjects at each time point were plotted (Supplementary Fig. S12). Overall, the dissimilarity between the samples was high, with median values of approximately 0.8. Temporal analysis of the distribution of distance values demonstrated that the distance between the subjects increased over time; the lowest distance (0.77) was seen at day 0 and the highest distance (0.83) was seen at day 90. Thus, instead of converging, the microbiota of the subjects actually appeared to slightly diverge over time; however, the biological significance of a change in diversity from 0.77 to 0.83 units is unclear. Of note, this result was in contrast to the results obtained by visual analysis of the Bray–Curtis PCoA plot over time (Fig. 4); therein the day 90 samples clustered together, implying that they were more similar. The observed differences between these two analyses perhaps demonstrates one of the drawbacks of a purely visual analysis of a PCoA plot, which attempts to represent highly dimensional data in two dimensions.

**Figure 6:**
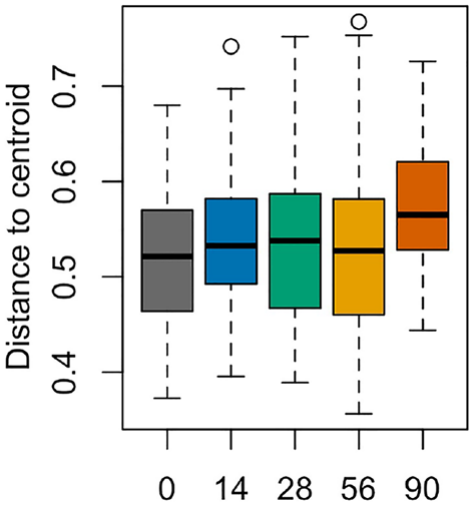
PERMDISP analysis to measure dispersivity. The homogeneity Bray–Curtis distances between the groups were measured using the PERMDISP^60^ analysis from the vegan package and graphed using *ggplot*. ANOVA was performed to test for differences, followed by pairwise comparisons with Tukey’s correction for multiple comparisons.

To further explore the apparent divergence in the microbiota, the dispersivity of the Bray–Curtis distances over time was measured using PERMDISP analysis^60^ (Fig. 6). This analysis measures the distance of each sample to the group centroid; larger distances from the centroid indicate less similarity in the group, whereas smaller distances from the centroid indicate more similarity in the group. The dispersivity of the samples was larger at day 90 than at other time points; as a factor, time was significant (ANOVA, *P* = 0.04057). Pairwise analysis determined that the difference was driven by the comparison of Day 0 to Day 90 (Tukey’s correction for multiple comparisons, *P* = 0.026883), the only significant comparison.

**Figure 7:**
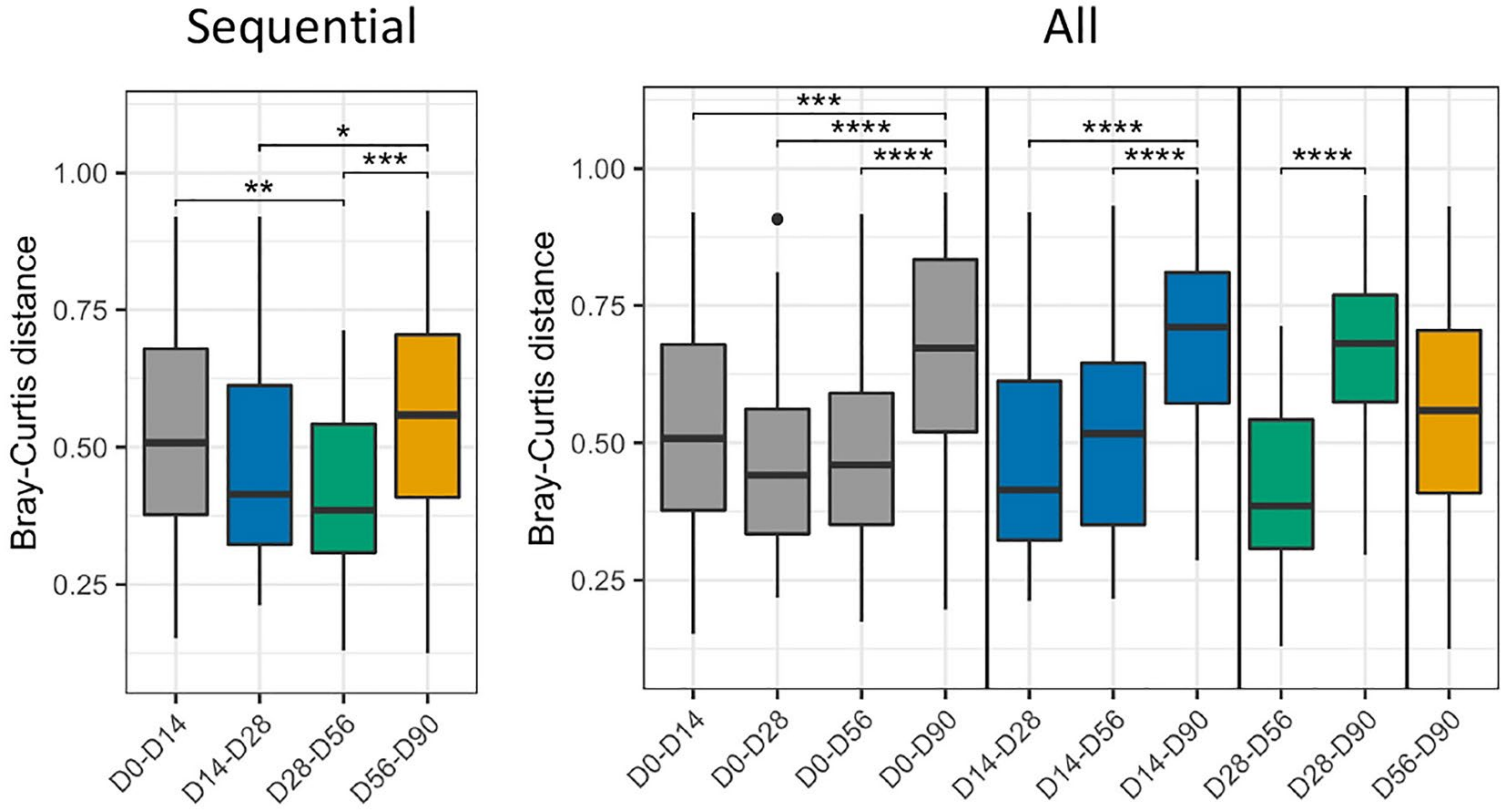
Stability and diversity of the microbiota. The changes in stability and diversity of the microbiota over the course of the study within subjects were evaluated using the Bray–Curtis distance. The pairwise time point-to-time point variation between same-subject samples was calculated and plotted as boxplots. Pairwise Wilcoxon tests with FDR correction for multiple comparisons were performed. Statistical significance is indicated by asterisks (*): *P < 0.05; **P < 0.01; ***P < 0.001; and ****P < 0.0001.

Lastly, to measure the stability of the microbiota over time, the pairwise time point-to-time point variation between same-subject samples was calculated (Fig. 7). This analysis measured the beta diversity of each subjects’ samples over time. Analysis of sequential time points (Day 0 to Day 14, Day 14 to Day 28, etc.) indicated that the dissimilarity of the microbiota between Day 0 and Day 14 was 0.51 units. The microbiota were more similar through Day 56 (0.41 units between Day 14 and Day 28 and 0.39 units between Day 28 and Day 56), and then most dissimilar between Day 56 and Day 90 (0.56 units). Analysis of all pairwise time points showed that the greatest dissimilarities were between Day 90 and any other day. These data suggest that the microbiota changed within the first 2 weeks, partly stabilized during training, and then shifted again before the last sample visit.

Taken together, the temporal analyses indicated that the microbiota of the trainees did change significantly over time. However, contrary to our original convergence hypothesis, the nasal microbiota of the subjects was more similar at day 0 than at day 90. The change in the microbiota of each subject was greatest at the beginning and end of the study, suggesting a biphasic evolution in the nasal microbiota. An early change occurred (between days 0 and 14), followed by some stability in the microbiota for several weeks (between days 14, 28, and 56), and finally another large shift occurred (between days 56 and 90). Thus, nasal microbiota evolution within the congregate setting imposed by military training is a complex process that appears to be affected by numerous factors.

## Discussion

To investigate the changes in the nasal microbiota that occur during initial military training, herein, we longitudinally assessed 53 Army trainees from a single platoon at Fort Benning, Georgia. Consistent with previous studies that examined the composition of the nasal microbiome in predominantly healthy individuals, the nasal microbiota of the cohort was dominated by 3 phyla: Actinobacteria, Firmicutes, and Proteobacteria^8,16,41,61,62^. Similarly, the prevalence of *S. aureus* nasal colonization of the trainees was comparable to previous reports^16,35,53,54^, with 39.2% of trainees not colonized, 43.1% of subjects intermittently colonized, and 17.6% of individuals persistently colonized (Fig. 1). As expected^63^, the number of *S. aureus* positive cultures increased from 22.6% of the recruits on day 0 to 37.2% of the trainees on day 90. Interestingly, of the two subjects that developed purulent SSTI, neither one was colonized by *S. aureus* in the nares at any of the sampled timepoints. This was a surprise since *S. aureus* nasal colonization is a risk factor for SSTI development^35,37,38^. It is possible that the congregate setting and outdoor activities engaged in by these subjects contributed to the development of the SSTI without a prior nasal colonization event. Alternatively, transient nasal colonization may have occurred outside of the obtained samples.

The microbiota of the individual trainees appeared strongly linked to *S. aureus* culture results and colonization status. *S. aureus* positive samples and samples from persistently colonized individuals contained similar genera, while samples that were negative for *S. aureus* and samples from non-colonized individuals contained a higher representation of *Corynebacterium_1* and the Neisseriaceae family (Supplementary Fig. S7B,C). Furthermore, we observed a significant increase in *Moraxella* at day 90 specifically among intermittently colonized subjects (Fig. 2, Supplementary Figs. S2, S4, and Supplementary Table S4). Of note, some members of *Moraxella* (*Moraxella catarrhalis*, specifically) are clinically relevant in respiratory disease^64,65^. The reasons why some individuals are persistently nasally colonized by *S. aureus* while others are intermittent carriers or persistent non-carriers remains unclear. However, it is clear that the presence of *S. aureus* within the nasal niche has a dramatic effect on the nasal microbiome. For example, in subjects that are persistently colonized by *S. aureus*, the microbiota is dominated by *S. aureus* (Supplementary Fig. S6). Thus, this bacterium may actively prevent colonization by other opportunistic bacteria. Similarly, in subjects that are persistent non-carriers of *S. aureus*, perhaps the dominant microbiota is able to prevent *S. aureus* (and possibly other opportunistic bacteria) from colonizing. In line with this, perhaps the nasal microbiome of intermittently colonized individuals represents a less robust microbiota that is unable to prevent temporary colonization by *S. aureus*, and, in the case of the trainees described here, *Moraxella* (Supplementary Fig. S6). This fluctuation may extend to other opportunistic pathogens, preventing or permitting colonization.

A temporal analysis of the stability of the microbiota revealed noteworthy shifts in composition at two distinct time points. The first change appeared to occur between day 0 and day 14, with the microbiota of the individual subjects then remaining stable for several weeks. Another large shift in the trainees’ microbiota then transpired between day 56 and day 90. These particular shifts could be related to activities in which the recruits were engaged during those specific time periods. For example, during week 12, the Infantry training cycle culminates in a week-long field training exercise, wherein soldiers conduct extensive training activities in austere environments and sleep in tents for 7 days before returning to the barracks. Of note, several of the genera identified in the LEfSe analysis are typically located in environmental samples, including soil and water^66–71^. Detecting these genera in the nares of trainees is not surprising given the amount of time trainees spend outside and the nature of their training that requires intense interaction with their environment.

Due to the small size of the cohort, some metadata could not be reliably included in tests, including ethnicity and race. Additionally, while the contribution of geographic origin to the microbiota is supported elsewhere^3,5–7^, our observations were complicated by the uneven number of subjects from each geographic region. Our cohort included 12 subjects from the Midwest, 10 from the Northeast, 26 from the South, and only 5 from the West. Furthermore, the assignment of geographic origin was based on the last permanent address provided by each subject in the study intake questionnaire; thus, the amount of time lived at that address and any gaps in which the subject had lived elsewhere were not captured. Consequently, the geographic location that potentially contributed to the nasal microbiome of a given individual may not be the same as the geographic region assigned through the intake questionnaire. Even given these limitations, the individuals’ microbiota still displayed some clustering based on geographic assignment when beta diversity was analyzed.

LEfSe analysis revealed important connections involving the *Staphylococcus* and *Corynebacterium* genera. Unsurprisingly, *Staphylococcus* was highly represented in individuals that were positive for *S. aureus* culture and in samples from persistently colonized individuals. In contrast, *Corynebacterium_1* was more highly represented in samples from trainees that were negative for *S. aureus* culture and in non-carriers of *S. aureus* (Supplementary Fig. S7). These data are consistent with our previous nasal microbiome studies that highlight an inverse relationship between colonization with *Corynebacterium* species and the presence of *Staphylococcus* species^41,44^. Furthermore, these data are in agreement with in vitro mechanistic studies that have shown the ability of *Corynebacterium* to kill pathogenic *S. aureus*^14–16^. If these in vitro results actually represent what happens within the nasal environment, this would explain why individuals carrying high levels of *Corynebacterium_*1 tend to be *S. aureus* negative or non-carriers. Similarly, it is worth noting that clinical studies show that direct introduction of *Corynebacterium* species into the nasal cavity can eradicate the *S. aureus* that is colonizing this niche^72^. Of note, the LEfSe analysis based on geographic origin revealed the differential representation of *Corynebacterium_1* in trainees from the Midwest (Supplementary Fig. S7). In addition, the trainees from this region had fewer *S. aureus-*positive cultures than trainees from the other regions; after removal of the two individuals that did not complete all study visits, 20% of samples obtained from those from the Midwest were positive, while 26% were positive from those from the Northeast, 31% from those from the South and 60% from those from the West (Supplementary Fig. S10). Given that nasal colonization with *S. aureus* is a risk factor for SSTI development, this leads to the intriguing possibility that those from the Midwest may be less likely to develop SSTI. To this end, future studies will address the contribution of this and other factors to SSTI development within military trainees.

To complete our longitudinal analysis of the nasal microbiota of platoon members, we analyzed the differences between the individuals’ microbiota at the start of the study and at the end of the study. We expected to see convergence of the microbiota similar to that observed in a study of cohabitating cadets at the U.S. Air Force Academy^23^. However, we did not find convergence among the microbiota and indeed noted divergence over time. Interestingly, the microbiota of the individual trainees appeared more similar at day 0 than at day 90. Our contrasting observation may result from a difference in the activities of this cohort, especially the outdoor activities performed by the platoon analyzed in this study.

## Conclusions

The congregate setting encountered during military training provides numerous, unique environmental and personal interactions for trainees. As such, the numerous encounters with microbes may provide an assault on the existing microbiota of the trainee. Indeed, the longitudinal investigation of the evolution of nasal microbiota presented here reveals a complex process that is affected by numerous factors. The stability of the nasal microbiota varied dramatically among the individuals, and the major driver of nasal microbiota stability appears to be the geographic origin of the trainees. Additionally, the stability of the nasal microbiota seems to correlate with *S. aureus* colonization status; however, the exact nature of the association between *S. aureus* colonization and nasal microbiota stability remains unclear. Indeed, a lack of stability within the nasal microbiota may facilitate colonization by *S. aureus*. These results suggest that broad-spectrum efforts to prevent or eradicate *S. aureus* colonization and subsequently minimize SSTIs within military settings may not be an effective approach. In contrast, a more individualized approach may ultimately increase the efficacy of these attempts to reduce the burden of SSTIs within the Military Health System.

## Methods

### Subject recruitment

A longitudinal cohort study was conducted, as described in detail elsewhere^52^. Trainees from the US Army Infantry at Fort Benning, Georgia, were recruited. The company commenced a 14-week training cycle in September 2015. Infantry training companies are composed of approximately 200 trainees, further segregated into four platoons. Samples from 53 trainees from a single platoon were analyzed for this longitudinal study. Among these individuals, 2 subjects developed a purulent SSTI: Subject 2013 on day 94; and Subject 2197 on day 22. The study was approved by the Uniformed Services University Institutional Review Board (IDCRP-090) and was conducted in accordance with the relevant guidelines and regulations of the institution. Informed consent was obtained from all study participants.

### Sample collection

Paired nasal swabs were obtained upon arrival (day 0) and at each study visit (day 14, 28, 56, and 90) by study staff for a total of five study visits. At the initial study visit, trainees completed questionnaires for demographic and MRSA risk factor information. From each set of paired nasal swabs, one swab was utilized to determine the presence of *S. aureus* by culture at the Benning Martin Army Community Hospital clinical microbiology laboratory as previously described^73^. The second swab was shipped to Uniformed Services University in Bethesda, MD for DNA extraction and microbiome analysis. Swabs were transported on dry ice and stored at − 80 °C until extraction, as described below.

*S. aureus* colonization status was defined based on the percent of study visits at which a subject was positive by culture, as follows: persistently colonized (≥ 80% positive); intermittently colonized (≥ 20% and < 80% positive); and not colonized (0% positive). Two subjects did not complete the five study visits, and no colonization status was assigned.

### DNA extraction

DNA was extracted from the nasal swabs using the PowerSoil-htp kit (MoBio), which combines chemical and mechanical lysis. A total of 261 sample swabs were arrayed across three 96-well plates. To extract the DNA from the sample, the swab head was broken into the 96-well plate. The location within the plates was randomized, with the exception that no subject could have all five samples in a single 96-well plate to minimize any possible plate-to-plate confounders. As a positive DNA extraction control, a mock microbial community (HM-280, BEI resources) was extracted in parallel. For this, approximately 1 × 10^8^ cells were added to one empty well within each 96-well plate, for a total of three positive controls. As a negative DNA extraction control, eight wells (3 in the first and second plate, and 2 in the third plate) were left empty. The manufacturer’s instructions were used with the following adaptations. PowerSoil bead solution and Solution C1 were mixed, transferred to each well of the plate, and incubated at 65 °C for 15 min prior to mechanical lysis. DNA was eluted from the silica membrane using 100 µL nuclease-free water (IDT).

### Microbiome library preparation

The V1–V3 region of the bacterial 16S rRNA gene was amplified using primers modified to include Illumina adapters, 27F (5′-AGAGTTTGATCCTGGCTCAG) and 534R (5′-ATTACCGCGGCTGCTGG) as previously described^74–76^. The following PCR conditions were used: 2.5 μL 10 × PCR Buffer, 4 μL dNTP Mix, 0.25 μL Takara LA Taq Polymerase (Clontech), 1 μL 27F (10 μM), 1 μL 534R (10 μM), 14.25 μL PCR Water (Qiagen), and 2.0 μL DNA. Reactions were performed in duplicate for 30 cycles, combined, purified using Agencourt AmpureXP (Beckman Coulter), and quantified using Quantifluor dsDNA Kit (Promega). Equivalent amounts of amplicons were pooled together, purified with MinElute PCR purification kit (Qiagen), and sequenced on an Illumina MiSeq along with the positive and negative sequencing controls. Due to a primer synthesis error, eleven of the patient samples yielded no reads. Thus, these samples were separately reamplified with corrected primers and sequenced. The resulting data were combined with the sequencing data from the other samples and then analyzed as described below.

### Sequencing analysis

V1–V3 16S rRNA amplicons were sequenced (2 × 300 bp MiSeq read pairs) and demultiplexed. Read pairs were quality trimmed using trimBWAstyle.pl (-q 20), trimmed of primers using tagcleaner.pl (v 0.16; -tag1 ATTACCGCGGCTGCTGG -tag2 AGAGTTTGATCCTGGCTCAG), filtered to remove short reads (< 246 nt) and merged using FLASH (v 1.2.11; default parameters)^55^.

The 16S rRNA gene sequences were processed using DADA2 v. 1.10.1^56,57^. FilterAndTrim was executed using default parameters for maximum N (maxN), truncation after a specific quality score (truncQ), and removal of phiX; in addition, reads were of length less than 400 base pairs were removed (minLen), and the maxEE score was set to 2. The error model was performed using 2 × 10^8^ bases, with multithread set to true. The data were dereplicated using derepFastq, and this dereplicated dataset was used for the sample inference algorithm. As the reads had already been overlapped, the step to merge forward and reverse reads was skipped. Chimeric sequences represented less than 3% of the amplicon sequence variants (ASVs), and were removed. Taxonomy was assigned using the Silva database (v132), with the optional species assignment.

Processing and analysis were performed using Phyloseq^77^. Taxonomic filtering was performed to remove the following: ASVs from five phyla that were represented with two or fewer ASVs (Deferribacteres, Dependentiae, Lentisphaerae, Nitrospirae, and Rokubacteria); ASVs from one eukaryotic phylum (Euglenozoa, 10 ASVs); and ASVs that did not classify to a phylum (180 ASVs). After taxonomic analysis, the positive and negative control samples (6 and 11 samples, respectively) were removed from the dataset for the downstream analyses. Post-DADA2 processing, 3 samples contained less than 1000 reads, and were also removed (Subject 2014 Day 28, Subject 2149 Day 28, and Subject 2198 Day 90).

Bar plots of percent abundance of phyla or genera were generated by agglomerating ASVs to the phylum or genus taxonomic level, keeping all taxa that classified to “NA” (NArm = FALSE), transforming read counts to relative abundance, and melting the phyloseq object into a matrix. Agglomerated phyla or genera that represented < 1% of reads at any time point were merged together using the summarize function of dplyr, as “Other”. Scatterplots of phyla or genera were generated using the agglomerated datasets. Alpha diversity (observed ASVs, Shannon diversity index, and Inverse Simpson index) and beta diversity (Bray–Curtis) were measured using *phyloseq*. Bray–Curtis distances were ordinated by principal co-ordinate analysis (PCoA) using *phyloseq*, and graphed using *phyloseq* and *ggplot*. All remaining graphs were generated using *ggplot*. Where shown, 95% confidence ellipses were plotted using stat_ellipse (*ggplot*). Graphs were arranged into grids using plot_grid (*cowplots*).

LEfSe analysis^58^ was performed using the Galaxy web application (https://huttenhower.sph.harvard.edu/galaxy). For each metadata set analyzed, the abundance of each agglomerated genus was uploaded as tabular data. Differences in abundance were tested using Kruskal–Wallis (*P* < 0.01) with the less strict one-against-all analysis. Differential features of 2 LDA were plotted.

### Statistics

Statistical analyses were conducted using R. Differences in abundance of genera or phyla were tested using one-way ANOVA across a single grouping or using two-way ANOVA using time and another grouping. Normality of alpha diversity measures was determined using the Shapiro–Wilk test, in which only the Shannon index was found to be normal (W = 0.99161, P = 0.1505). Subsequently, parametric (ANOVA and Student’s *t*) or non-parametric (Kruskal–Wallis Rank Sum and Wilcoxon Rank Sum) tests were employed. Where significant associations were found, pairwise Student’s *t* and pairwise Wilcoxon Rank Sum tests were employed, with Benjamini and Hochberg method (FDR) to correct for multiple comparisons. The *vegan* package was used to test one-way differences in beta diversity (PERMANOVA), for analysis of similarity (ANOSIM), and for beta dispersivity (PERMDISP) with one-way ANOVA to test for differences. Two-way differences in beta diversity were tested using pairwise PERMANOVA, using an adaptation of *vegan*’s adonis function^78^. Differences in Bray–Curtis distances were performed by pairwise Wilcoxon test with FDR correction for multiple comparisons.

### Ethics approval and consent to participate

The study was approved by the Uniformed Services University Institutional Review Board (IDCRP-090).

## Supplementary Information

Supplementary Figure S1.

Supplementary Figure S2.

Supplementary Figure S3.

Supplementary Figure S4.

Supplementary Figure S5.

Supplementary Figure S6.

Supplementary Figure S7.

Supplementary Figure S8.

Supplementary Figure S9.

Supplementary Figure S10.

Supplementary Figure S11.

Supplementary Figure S12.

Supplementary Table S1.

Supplementary Table S2.

Supplementary Tables.

## Data Availability

The datasets generated and analyzed during the current study are available in the NCBI under BioProject ID: PRJNA780476 [https://dataview.ncbi.nlm.nih.gov/object/PRJNA780476].
