# Supplementary figures and images for: Nasal microbiota evolution within the congregate setting imposed by military training
Source: Sci Rep. 2022 Jul 7;12:11492. doi: 10.1038/s41598-022-15059-z (PMC9263147; doi:10.1038/s41598-022-15059-z)

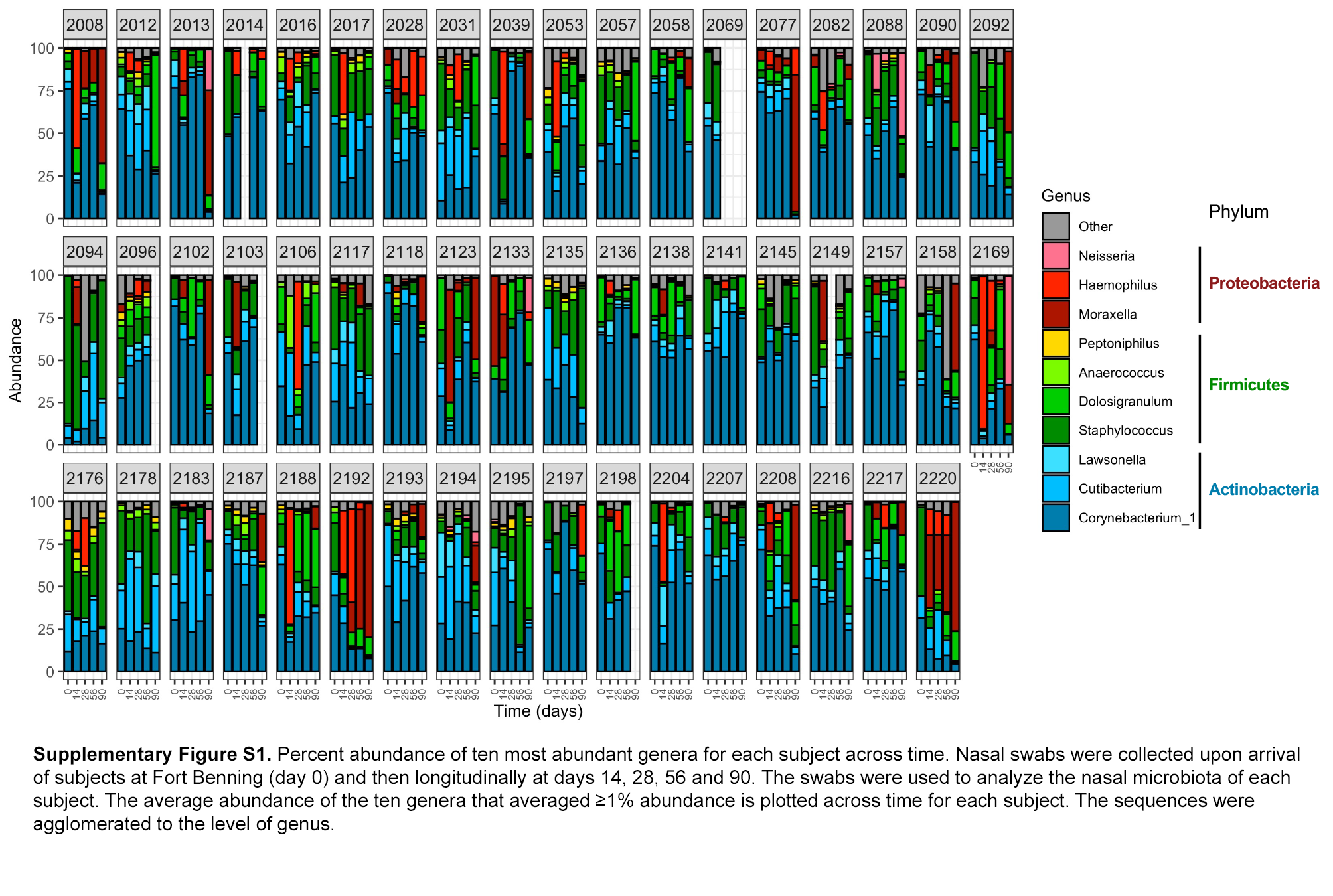

Supplement: Supplementary file 1 — Supplementary Figure S1. [file 41598_2022_15059_MOESM1_ESM.tif]

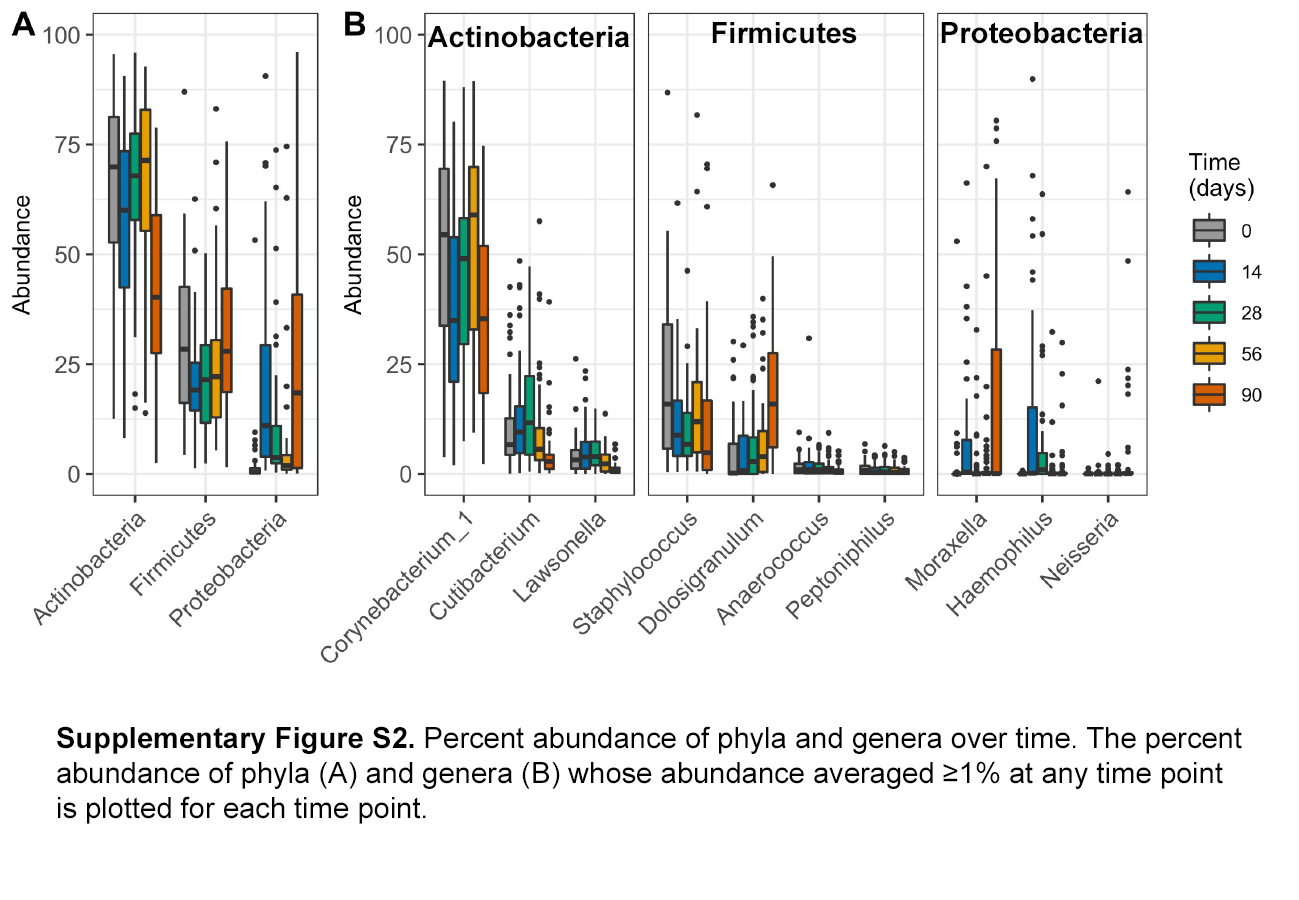

Supplement: Supplementary file 2 — Supplementary Figure S2. [file 41598_2022_15059_MOESM2_ESM.tif]

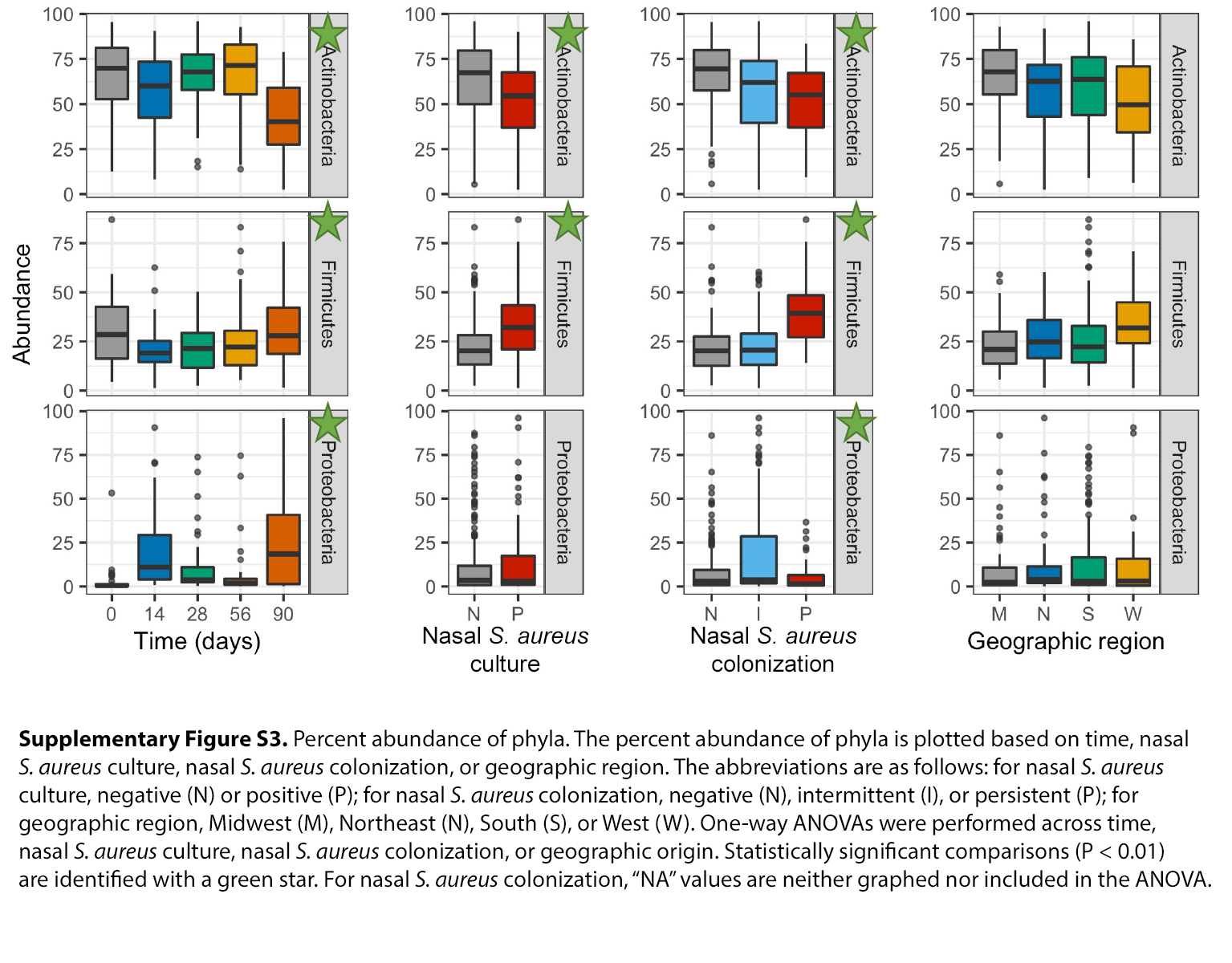

Supplement: Supplementary file 3 — Supplementary Figure S3. [file 41598_2022_15059_MOESM3_ESM.tif]

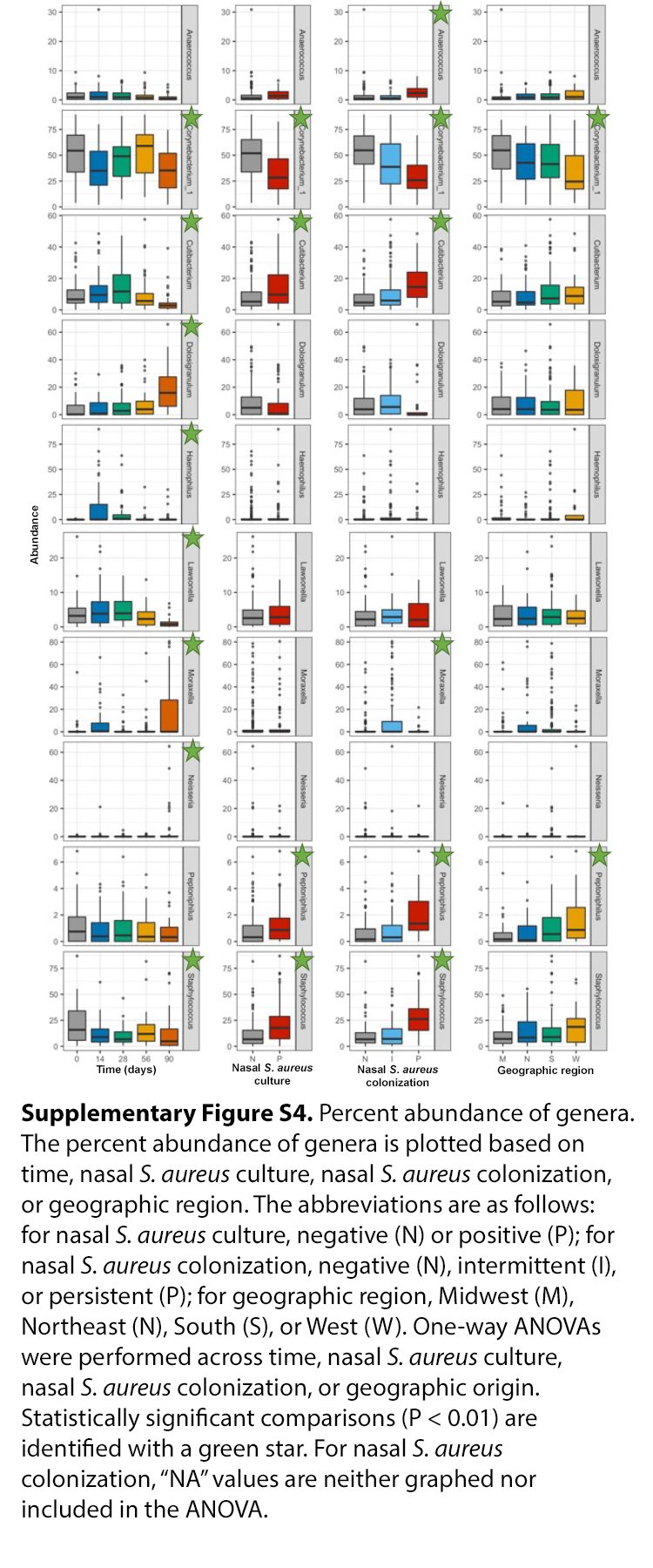

Supplement: Supplementary file 4 — Supplementary Figure S4. [file 41598_2022_15059_MOESM4_ESM.tif]

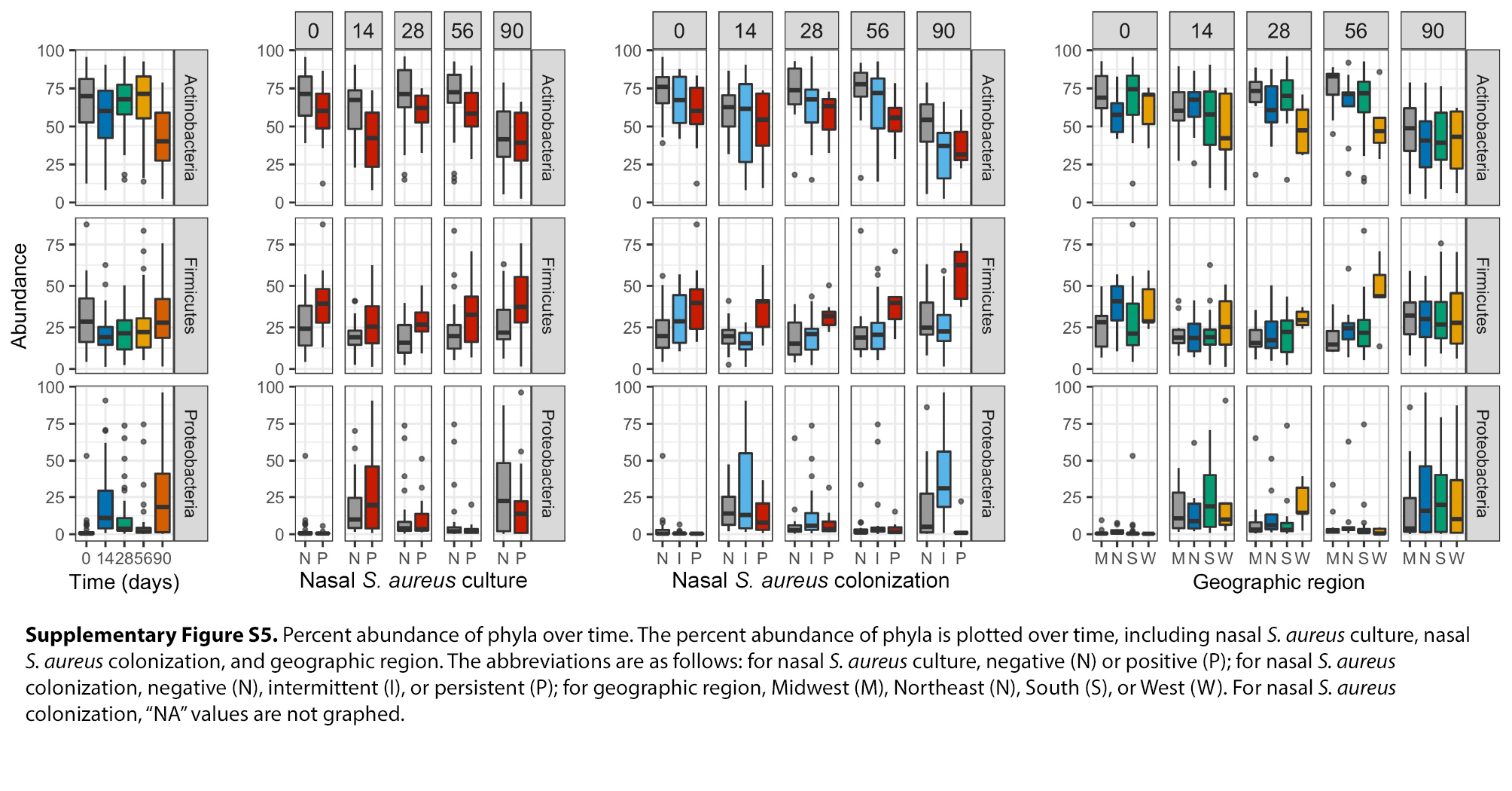

Supplement: Supplementary file 5 — Supplementary Figure S5. [file 41598_2022_15059_MOESM5_ESM.tif]

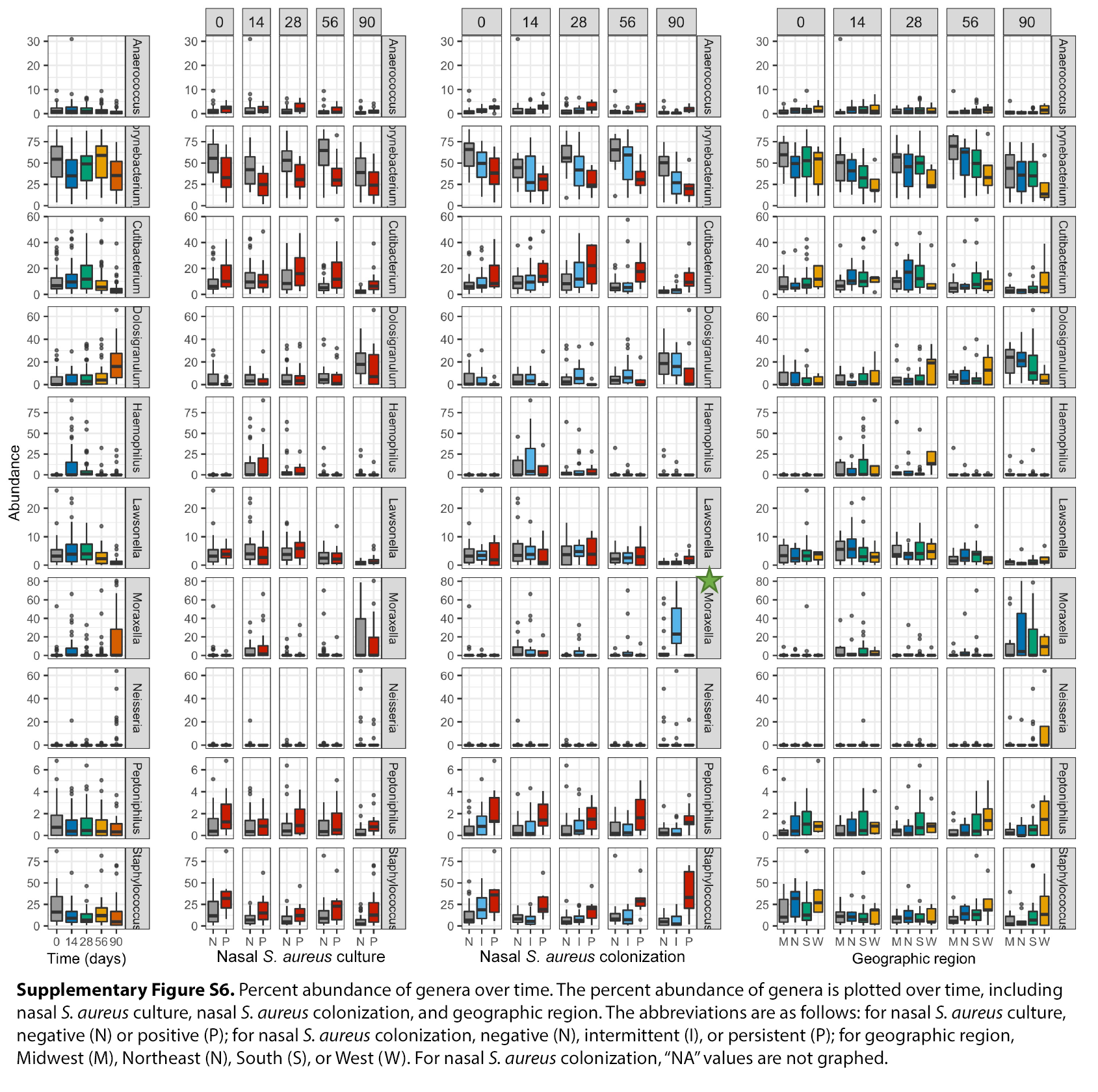

Supplement: Supplementary file 6 — Supplementary Figure S6. [file 41598_2022_15059_MOESM6_ESM.tif]

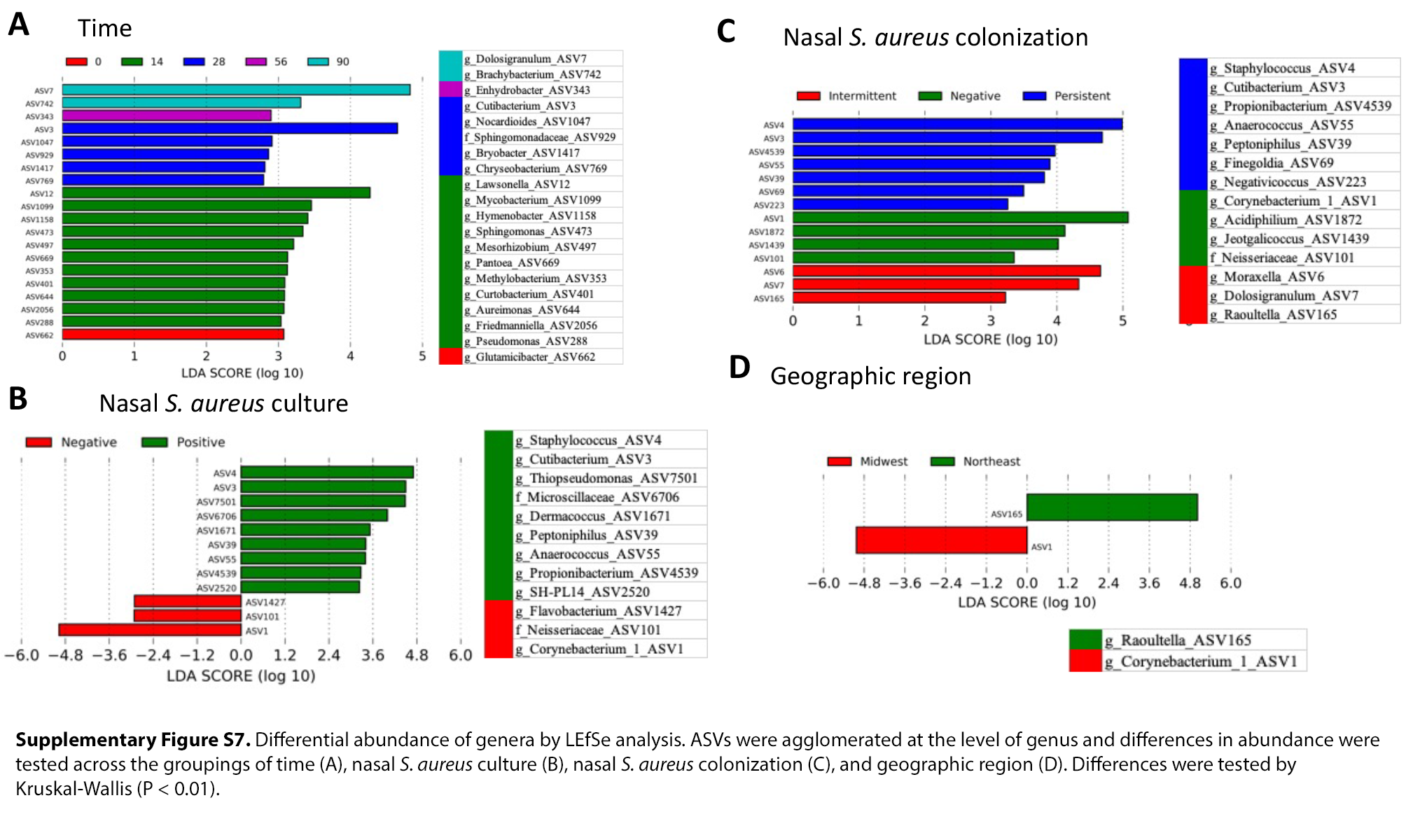

Supplement: Supplementary file 7 — Supplementary Figure S7. [file 41598_2022_15059_MOESM7_ESM.tif]

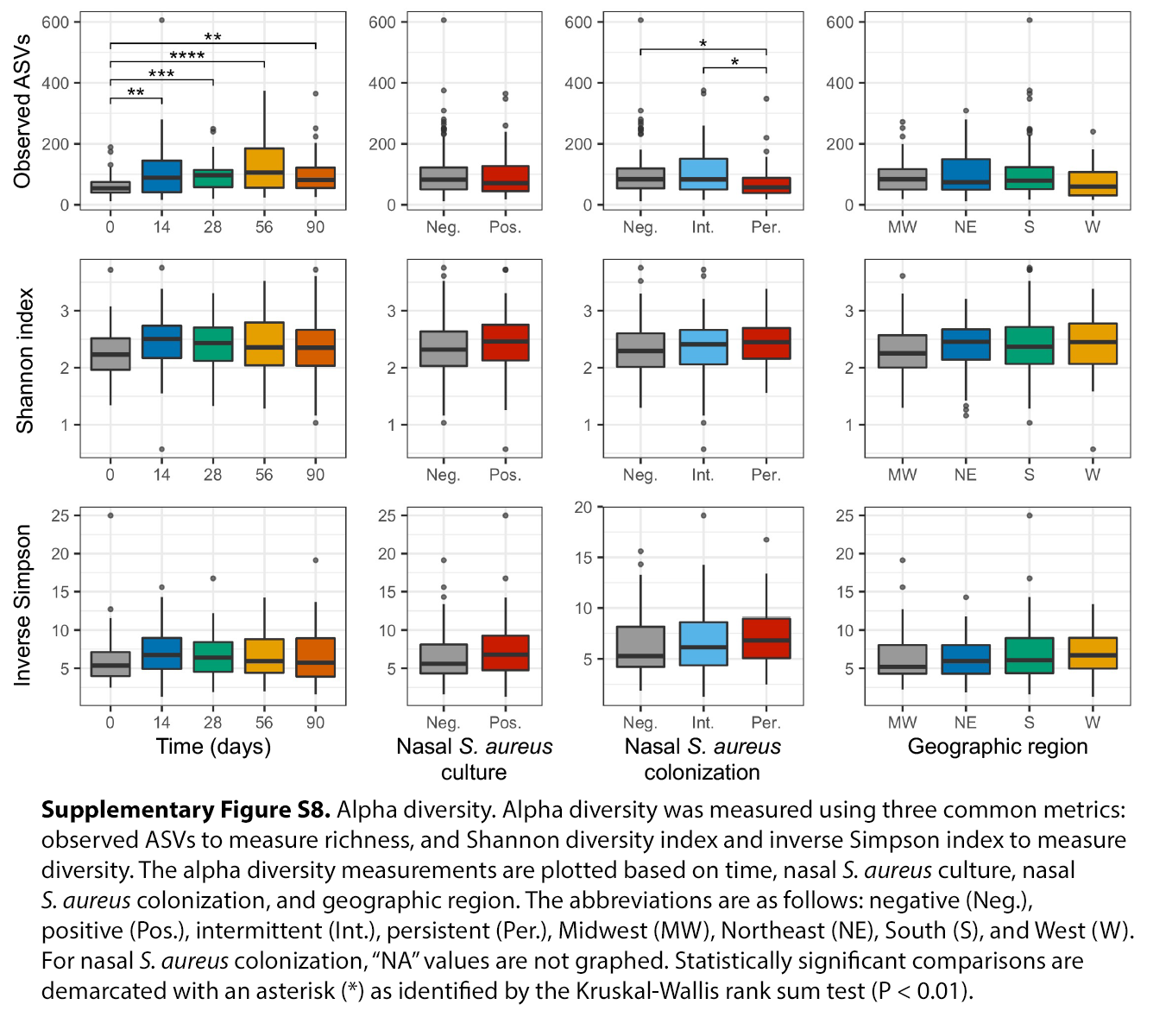

Supplement: Supplementary file 8 — Supplementary Figure S8. [file 41598_2022_15059_MOESM8_ESM.tif]

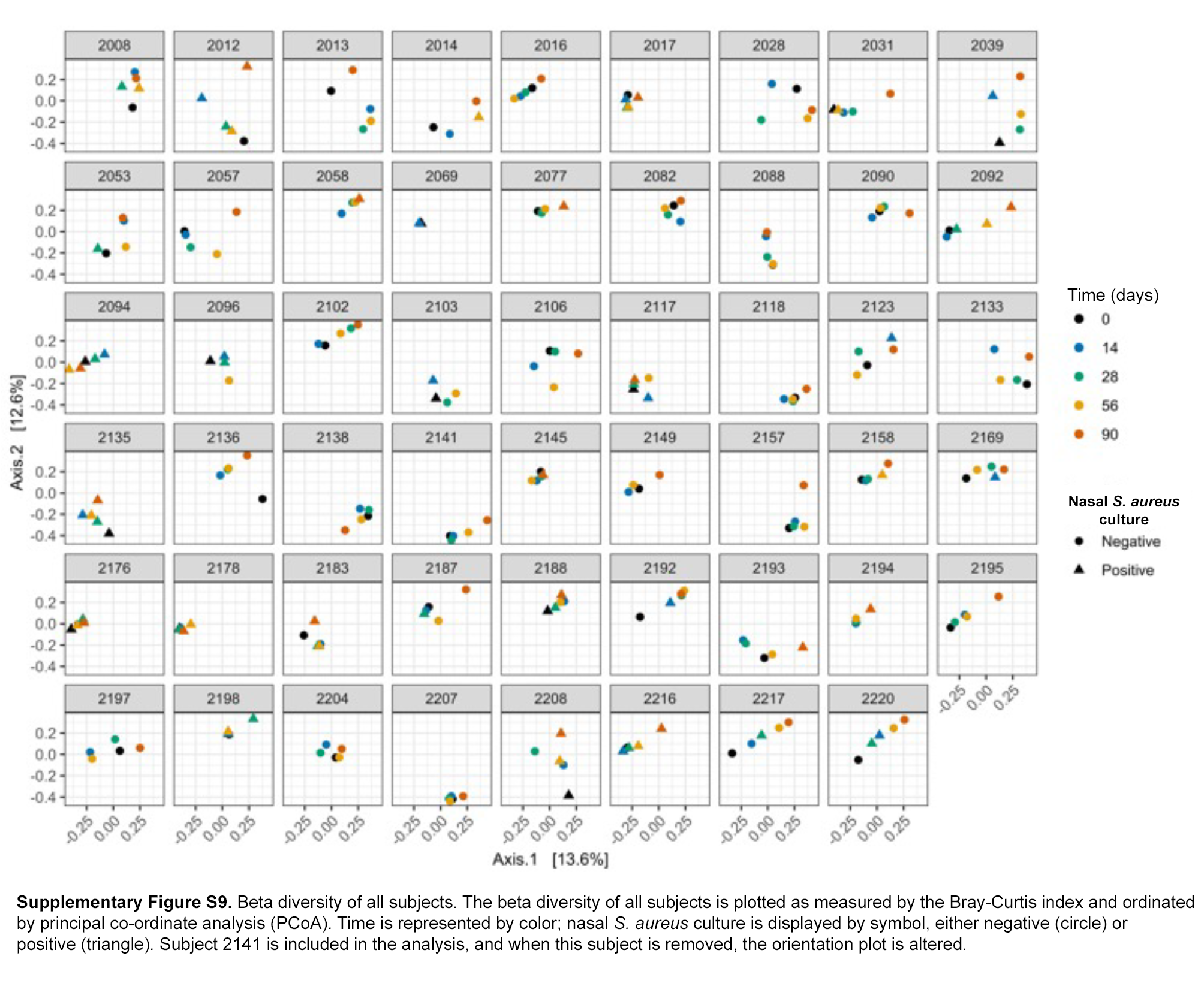

Supplement: Supplementary file 9 — Supplementary Figure S9. [file 41598_2022_15059_MOESM9_ESM.tif]

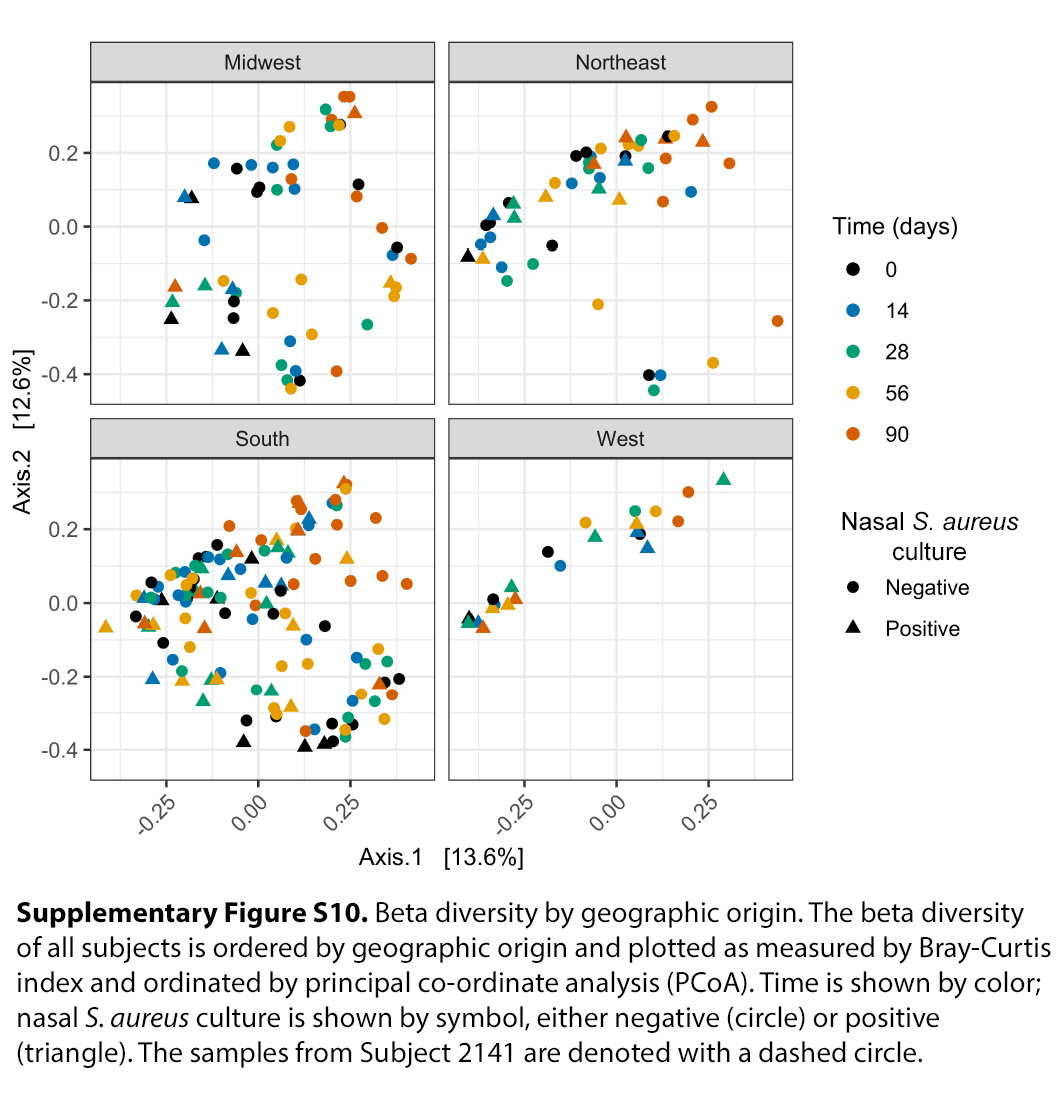

Supplement: Supplementary file 10 — Supplementary Figure S10. [file 41598_2022_15059_MOESM10_ESM.tif]

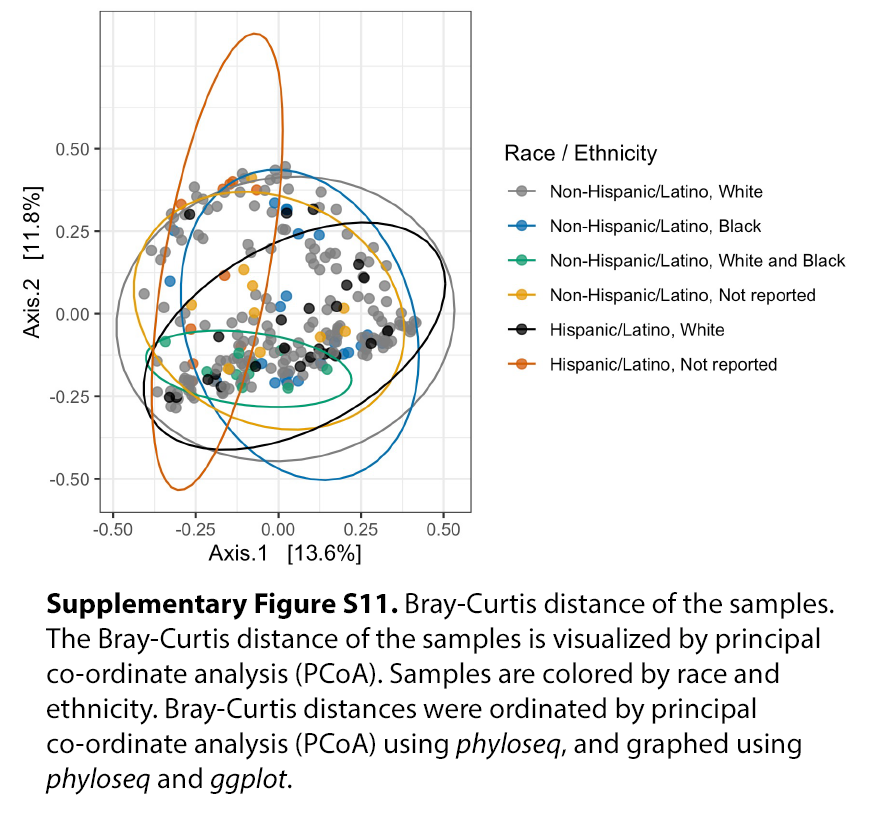

Supplement: Supplementary file 11 — Supplementary Figure S11. [file 41598_2022_15059_MOESM11_ESM.tif]

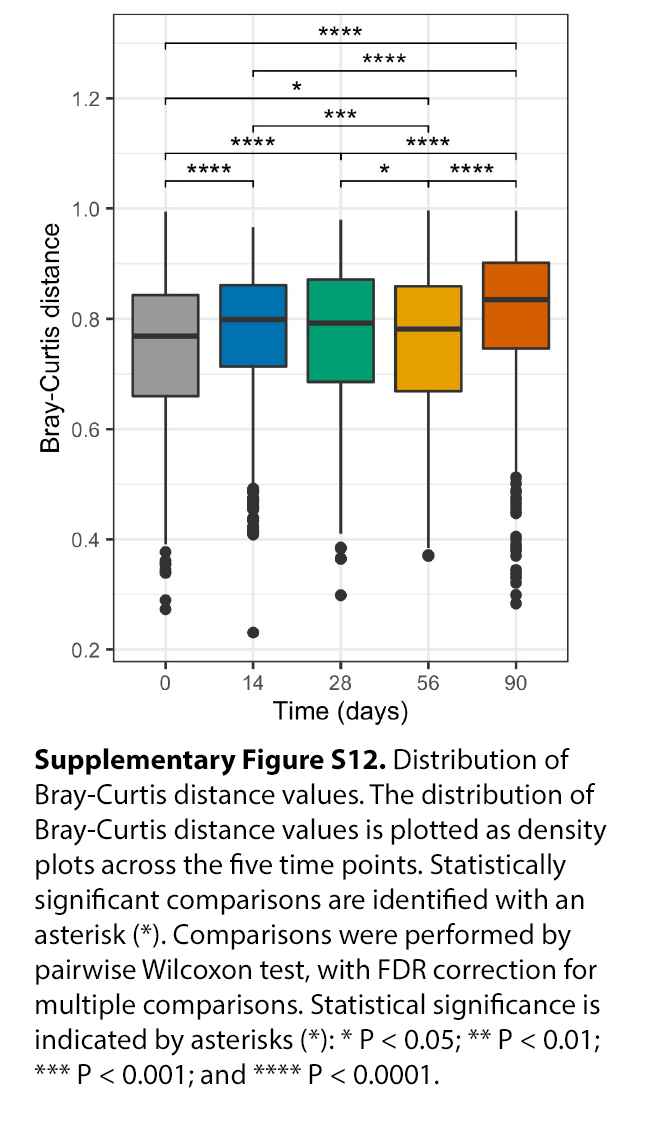

Supplement: Supplementary file 12 — Supplementary Figure S12. [file 41598_2022_15059_MOESM12_ESM.tif]
