# Supplementary material for: Nasal microbiota evolution within the congregate setting imposed by military training
Source: Sci Rep. 2022 Jul 7;12:11492. doi: 10.1038/s41598-022-15059-z (PMC9263147; doi:10.1038/s41598-022-15059-z)
Supplement: Supplementary file 15 — Supplementary Tables. [file 41598_2022_15059_MOESM15_ESM.docx]

**Supplementary Table S3.** Phylum abundance. One-way ANOVAs were performed across time, nasal *S. aureus* culture, nasal *S. aureus* colonization, or geographic origin. For nasal *S. aureus* colonization, subjects with “NA” status were removed prior to the test.

| Phylum | Parameter | Significance |
| --- | --- | --- |
| Actinobacteria | Time | 9.19e-12 *** |
| Actinobacteria | Nasal culture | 2.42e-05 *** |
| Actinobacteria | Nasal status | 0.000251 *** |
| Actinobacteria | Geographic region | 0.0636 |
| Firmicutes | Time | 0.000225 *** |
| Firmicutes | Nasal culture | 1.11e-07 *** |
| Firmicutes | Nasal status | 2.26e-11 *** |
| Firmicutes | Geographic region | 0.131920 |
| Proteobacteria | Time | 4.31e-10 *** |
| Proteobacteria | Nasal culture | 0.884 |
| Proteobacteria | Nasal status | 0.00068 *** |
| Proteobacteria | Geographic region | 0.744 |

*** *P* < 0.001.

**Supplementary Table S4**. Genera abundance. One-way ANOVAs were performed across time, nasal *S. aureus* culture, nasal *S. aureus* colonization, or geographic origin. For nasal *S. aureus* colonization, subjects with “NA” status were removed prior to the test.

| Genus | Parameter | Significance |
| --- | --- | --- |
| Corynebacterium_1 | Time | 6.35e-07 *** |
| Corynebacterium_1 | Nasal culture | 5.16e-11 *** |
| Corynebacterium_1 | Nasal status | 3.45e-10 *** |
| Corynebacterium_1 | Geographic region | 0.0217 * |
| Cutibacterium | Time | 5.45e-05 *** |
| Cutibacterium | Nasal culture | 3.93e-06 *** |
| Cutibacterium | Nasal status | 3.57e-06 *** |
| Cutibacterium | Geographic region | 0.486 |
| Lawsonella | Time | 1.59e-06 *** |
| Lawsonella | Nasal culture | 0.809 |
| Lawsonella | Nasal status | 0.9 |
| Lawsonella | Geographic region | 0.710 |
| Moraxella | Time | 3.12e-08 *** |
| Moraxella | Nasal culture | 0.943 |
| Moraxella | Nasal status | 0.000623 *** |
| Moraxella | Geographic region | 0.317 |
| Haemophilus | Time | 3.55e-07 *** |
| Haemophilus | Nasal culture | 0.844 |
| Haemophilus | Nasal status | 0.265 |
| Haemophilus | Geographic region | 0.353 |
| Neisseria | Time | 0.000351 *** |
| Neisseria | Nasal culture | 0.400552 |
| Neisseria | Nasal status | 0.778 |
| Neisseria | Geographic region | 0.252593 |
| Staphylococcus | Time | 0.000168 *** |
| Staphylococcus | Nasal culture | 8.36e-09 *** |
| Staphylococcus | Nasal status | 2.87e-13 *** |
| Staphylococcus | Geographic region | 0.384793 |
| Dolosigranulum | Time | 5.5e-11 *** |
| Dolosigranulum | Nasal culture | 0.155 |
| Dolosigranulum | Nasal status | 0.121 |
| Dolosigranulum | Geographic region | 0.592 |
| Anaerococcus | Time | 0.0786 |
| Anaerococcus | Nasal culture | 0.1483 |
| Anaerococcus | Nasal status | 0.00206 ** |
| Anaerococcus | Geographic region | 0.8593 |
| Peptoniphilus | Time | 0.173406 |
| Peptoniphilus | Nasal culture | 0.000876 *** |
| Peptoniphilus | Nasal status | 5.06e-08 *** |
| Peptoniphilus | Geographic region | 0.003782 ** |

* *P* < 0.05; ** *P* < 0.01; *** *P* < 0.001.

**Supplementary Table S5**. Abundance of phyla. Two-way ANOVAs were performed by time, and by a second group: nasal *S. aureus* culture, nasal *S. aureus* colonization, or geographic origin. For nasal *S. aureus* colonization, subjects with “NA” status were removed prior to the test.

| Phylum | First group | Second group | Pr (<F) |
| --- | --- | --- | --- |
| Actinobacteria | Time | Nasal culture | 0.525 |
| Actinobacteria | Time | Nasal status | 0.955 |
| Actinobacteria | Time | Geographic region | 0.8639 |
| Firmicutes | Time | Nasal culture | 0.937108 |
| Firmicutes | Time | Nasal status | 0.12 |
| Firmicutes | Time | Geographic region | 0.673564 |
| Proteobacteria | Time | Nasal culture | 0.147 |
| Proteobacteria | Time | Nasal status | 0.025306 * |
| Proteobacteria | Time | Geographic region | 0.90 |

* *P* < 0.05

**Supplementary Table S6**. Abundance of genera. Two-way ANOVAs were performed by time, and by a second group: nasal *S. aureus* culture, nasal *S. aureus* colonization, or geographic origin. For nasal *S. aureus* colonization, subjects with “NA” status were removed prior to the test.

| Genus | First group | Second group | Pr (<F) |
| --- | --- | --- | --- |
| Corynebacterium_1 | Time | Nasal culture | 0.849 |
| Corynebacterium_1 | Time | Nasal status | 0.911 |
| Corynebacterium_1 | Time | Geographic region | 0.98471 |
| Cutibacterium | Time | Nasal culture | 0.124 |
| Cutibacterium | Time | Nasal status | 0.97 |
| Cutibacterium | Time | Geographic region | 0.952116 |
| Lawsonella | Time | Nasal culture | 0.463 |
| Lawsonella | Time | Nasal status | 0.789 |
| Lawsonella | Time | Geographic region | 0.742 |
| Staphylococcus | Time | Nasal culture | 0.367791 |
| Staphylococcus | Time | Nasal status | 0.0509 |
| Staphylococcus | Time | Geographic region | 0.736716 |
| Dolosigranulum | Time | Nasal culture | 0.425 |
| Dolosigranulum | Time | Nasal status | 0.7324 |
| Dolosigranulum | Time | Geographic region | 0.179 |
| Anaerococcus | Time | Nasal culture | 0.5352 |
| Anaerococcus | Time | Nasal status | 0.96149 |
| Anaerococcus | Time | Geographic region | 0.9814 |
| Peptoniphilus | Time | Nasal culture | 0.57701 |
| Peptoniphilus | Time | Nasal status | 0.895 |
| Peptoniphilus | Time | Geographic region | 0.992987 |
| Moraxella | Time | Nasal culture | 0.321 |
| Moraxella | Time | Nasal status | 0.000109*** |
| Moraxella | Time | Geographic region | 0.996 |
| Haemophilus | Time | Nasal culture | 0.570 |
| Haemophilus | Time | Nasal status | 0.234 |
| Haemophilus | Time | Geographic region | 0.498 |
| Neisseria | Time | Nasal culture | 0.844498 |
| Neisseria | Time | Nasal status | 0.999473 |
| Neisseria | Time | Geographic region | 0.133300 |

*** *P* < 0.001

**Supplementary Table S7**. Measures of alpha diversity were tested for significance. One-way comparisons were tested across time, nasal *S. aureus* culture, nasal *S. aureus* colonization, or geographic origin. Alpha diversity metrics were number of observed ASVs, Shannon diversity index, and inverse Simpson index. The test performed for each comparison is listed.

| Alpha diversity metric | Parameter | Test (reported statistic)^#^ | Significance |
| --- | --- | --- | --- |
| Observed ASVs | Time | Kruskal-Wallis (P-value) | 7.237e-05*** |
| Observed ASVs | Nasal culture | Wilcoxon rank sum (P-value) | 0.5129 |
| Observed ASVs | Nasal status | Kruskal-Wallis (P-value) | 0.03305* |
| Observed ASVs | Geographic region | Kruskal-Wallis (P-value) | 0.4896 |
| Shannon index | Time | ANOVA (Pr>F) | 0.146 |
| Shannon index | Nasal culture | Welch Two Sample t-test (P-value) | 0.1613 |
| Shannon index | Nasal status | ANOVA (Pr>F) | 0.344 |
| Shannon index | Geographic region | ANOVA (Pr>F) | 0.693 |
| Inverse Simpson | Time | Kruskal-Wallis (P-value) | 0.3474 |
| Inverse Simpson | Nasal culture | Wilcoxon rank sum (P-value) | 0.07681 |
| Inverse Simpson | Nasal status | Kruskal-Wallis (P-value) | 0.122 |
| Inverse Simpson | Geographic region | Kruskal-Wallis (P-value) | 0.3585 |

* *P* < 0.05; *** *P* < 0.001

**^#^**The Shapiro test for normality was employed. The observed ASVs and inverse Simpson metrics were not normally distributed, while the Shannon index metric was normally distributed. The Kruskal-Wallis and Wilcoxon test were run for non-normally distributed data, and ANOVA and t-test were run for normally distributed data.

**Supplementary Table S8**. Bray-Curtis beta diversity. Bray-Curtis beta diversity distances were tested by pairwise PERMANOVA analysis for time, nasal *S. aureus* colonization, and geographic region. For nasal *S. aureus* colonization, subjects with “NA” status were removed. Pairwise PERMANOVA was performed using an adaptation of vegan’s adonis function [78], using 999 permutations.

| Time | Adjusted P-value |
| --- | --- |
| 0 vs 14 | 0.001** |
| 0 vs 28 | 0.2358 |
| 0 vs 56 | 0.9584 |
| 0 vs 90 | 0.001** |
| 14 vs 28 | 0.206 |
| 14 vs 56 | 0.001** |
| 14 vs 90 | 0.001** |
| 28 vs 56 | 0.9584 |
| 28 vs 90 | 0.001** |
| 56 vs 90 | 0.001** |
| **Nasal colonization** | **Adjusted P-value** |
| Intermittent vs Persistent | 0.0003*** |
| Intermittent vs Negative | 0.0003*** |
| Persistent vs Negative | 0.0003*** |
| **Geographic region** | **Adjusted P-value** |
| South vs Midwest | 0.0045** |
| South vs Northeast | 0.0006*** |
| South vs West | 0.0006*** |
| Midwest vs Northeast | 0.0006*** |
| Midwest vs West | 0.0006*** |
| Northeast vs West | 0.0022** |

** *P* < 0.01; *** *P* < 0.001
